# Mechanistic insights and translational perspectives on liver fibrosis through single-cell transcriptomics

**DOI:** 10.3389/fimmu.2026.1954491

**Published:** 2026-10-27

**Authors:** Weiwei Lu, Jun Xu, Kexin Wang, Yumu Chen, Alessandro Cherubini, Luca Valenti, Shusen Zheng, Zhengtao Liu

**Affiliations:** 1 Key Laboratory of Artificial Organs and Computational Medicine in Zhejiang Province, Shulan International Medical College, Zhejiang Shuren University, Hangzhou, China; 2 Shulan (Hangzhou) Hospital, Shulan International Medical College, Zhejiang Shuren University, Hangzhou, China; 3 Division of Hepatobiliary and Pancreatic Surgery, Department of Surgery, First Affiliated Hospital, School of Medicine, Zhejiang University, Hangzhou, China; 4 Precision Medicine Lab, Biological Resource Center and Transfusion Medicine, Fondazione Istituto di Ricovero e Cura a Carattere Scientifico Ca’ Granda Ospedale Maggiore Policlinico, Milano, Italy; 5 Department of Pathophysiology and Transplantation, Università degli Studi di Milano, Milano, Italy; 6 National Health Commission of the People's Republic of China Key Laboratory of Combined Multi-Organ Transplantation, Key Laboratory of the Diagnosis and Treatment of Organ Transplantation, First Affiliated Hospital, School of Medicine, Zhejiang University, Hangzhou, China; 7 Key Laboratory of Organ Transplantation, Zhejiang Province, First Affiliated Hospital, School of Medicine, Zhejiang University, Hangzhou, China

**Keywords:** B lymphocytes, cell trajectory analysis, cell–cell communication, hepatic stellate cells, liver fibrosis, liver sinusoidal endothelial cells, macrophages, single-cell transcriptomics

## Abstract

Liver fibrosis is a defining feature of chronic liver diseases and can progress to cirrhosis and liver failure. Single-cell RNA sequencing (scRNA-seq) enables high-resolution analysis of cellular heterogeneity and multicellular interactions during fibrogenesis. In this narrative review, we analyzed published single-cell transcriptomic studies from experimental models and human cohorts, focusing on cell-type–specific programs, cell-state trajectories inferred by Monocle, and intercellular communication networks predicted using CellChat and CellPhoneDB. Hepatic stellate cells (HSCs) consistently emerged as the primary extracellular matrix–producing cells, with conserved activation signatures involving collagen synthesis, Phosphatidylinositol 3-kinase–Protein kinase B (PI3K–AKT) signaling, and matrix organization. Macrophage analyses identified fibrosis-associated subsets, including CD9^+^ and TREM2^+^ scar-associated macrophages (SAMs), which regulate inflammation, angiogenesis, and stromal remodeling. Liver sinusoidal endothelial cells undergo capillarization and transcriptional reprogramming linked to WNT signaling and antigen presentation. B cells exhibit activation signatures related to B-cell receptor signaling. Trajectory analyses infer potential cell-state transitions based on transcriptional similarity from quiescent to pathogenic states, including stepwise HSC activation and monocyte-to-macrophage differentiation. Cell–cell communication analysis highlights HSC–macrophage interactions, with key axes such as predicted Chemokine ligand 2–chemokine receptor 2 (CCL2–CCR2) and transforming growth factor-β–transforming growth factor-β receptor (TGFβ–TGFβR) signaling interactions that may contribute to fibrogenic communication networks. These findings define conserved multicellular programs and support integrated multi-omics approaches for biomarker discovery and therapy.

## 1 Introduction

Liver fibrosis represents a pathological wound-healing response to prolonged hepatic injury and constitutes the pivotal intermediate stage leading to cirrhosis, hepatic decompensation and hepatocellular carcinoma (1). It is characterized by progressive extracellular matrix (ECM) accumulation and structural remodeling that eventually disrupt normal liver architecture and impair organ function (2). According to recent epidemiological analyses, chronic liver disease—including fibrosis and its advanced stages—accounts for over 1.5–2 million deaths annually worldwide, ranking among the top causes of mortality (3). In recent decades, metabolic dysfunction–associated steatohepatitis (MASH), viral hepatitis B and C (HBV, HCV), alcohol-related liver disease and cholestatic disorders have emerged as major etiological contributors (4). Notably, the global shift toward obesity and metabolic syndrome drives a continuous increase in fibrosis prevalence, particularly in regions where antiviral treatments have already reduced hepatitis-related complications (5). This epidemiological transition highlights the urgent need for mechanistic insight and the development of targeted antifibrotic therapies, as effective treatment options remain limited across fibrosis etiologies (6). Resmetirom has demonstrated histological benefit in selected patients with noncirrhotic NASH and fibrosis (7).

Liver fibrosis arises from complex interactions among multiple hepatic cell populations and is governed by intricate molecular pathways. Hepatic stellate cells serve as the principal matrix-producing cells and transdifferentiate into myofibroblast-like phenotypes under chronic injury. Injured hepatocytes exhibit metabolic reprogramming and generate paracrine stress signals that activate hepatic stellate cells (HSCs) and other non-parenchymal cells (8, 9). Liver sinusoidal and vascular endothelial cells lose fenestrations and shift toward capillarization, contributing to impaired perfusion and inflammatory cell recruitment (10). Resident Kupffer cells, monocyte-derived macrophages and specific lymphoid cell subsets secrete profibrogenic mediators such as TGF-β, Platelet-derived growth factor (PDGF), WNT ligands and IL-1β, amplifying ECM deposition, tissue contraction and remodeling (11, 12). In parallel, fibro-resolving immune responses remain insufficient to counterbalance progressive fibrogenic activity during chronic injury (13). These cellular and molecular events reshape the hepatic microenvironment and determine fibrosis severity and reversibility.

Traditional investigative approaches—including histopathology, primary cell culture models, bulk transcriptomics, proteomics and metabolomics—have contributed substantially to the understanding of fibrosis pathophysiology and facilitated the identification of key signaling pathways and potential therapeutic targets (14–16). However, these techniques analyze composite tissue samples, averaging molecular information across diverse cell types and obscuring rare or transient cellular states (17). They fail to delineate cell subtype-specific transcriptional programs, dynamic activation trajectories or ligand–receptor interactions that underpin intercellular communication in the fibrotic niche (18). Consequently, essential regulatory cell populations associated with disease progression or resolution may remain undetected, and mechanistic insights derived from bulk datasets cannot be directly leveraged for cell-specific therapeutic strategies (19, 20).

Single-cell and single-nucleus RNA sequencing (sc/snRNA-seq) provide high-resolution transcriptomic profiling at the cellular level, enabling the identification of distinct cellular subsets, characterization of activation dynamics and reconstruction of cell-state transitions (21, 22). In liver fibrosis research, scRNA-seq has revealed previously unknown scar-associated macrophages (SAMs; e.g., TREM2^+^CD9^+^), heterogeneous HSC and perivascular mesenchymal cell populations, specialized endothelial cell subsets involved in vascular remodeling and distinct lymphocyte states with regulatory or profibrotic potential (23). Furthermore, trajectory inference, RNA velocity analysis and spatial transcriptomic integration allow mapping of cellular progression pathways and microenvironmental positioning in both human samples and animal models. These capabilities generate mechanistic insights directly relevant to therapeutic target prioritization, biomarker development and potential patient stratification strategies (24, 25).

This narrative review synthesizes current single-cell transcriptomic discoveries in liver fibrosis. We examine cellular heterogeneity and transcriptional reprogramming in major fibrogenic and immunomodulatory cell populations—including HSCs, macrophages, liver sinusoidal endothelial cells (LSECs), and B-lymphocytes—by integrating evidence from human studies and complementary animal models. This evidence delineates the profound remodeling of cellular composition in fibrotic livers characterized by expansion of pathogenic populations and loss of homeostatic states. We further summarize advances in Monocle-based pseudotime analysis and intercellular communication mapping. These methods help reconstruct the dynamic evolution of fibrosis, including the activation of quiescent HSCs, differentiation of monocyte-derived macrophages, and the coordinated signaling interactions within the fibrotic niche. In particular, ligand–receptor–based analyses derived from CellChat and CellPhoneDB computational frameworks reveal multicellular communication networks that orchestrate inflammation, extracellular matrix deposition, and vascular remodeling. Pseudotime suggests cell-state relationships but does not establish lineage. By integrating these dimensions, single-cell transcriptomics provides a systems-level framework to define the cellular and molecular mechanisms underlying fibrogenesis, including cell-type–specific regulatory networks, spatiotemporal transitions of pathogenic cell states, and key signaling pathways driving disease progression. These insights not only enhance mechanistic understanding at the cellular level but also establish a theoretical foundation for biomarker discovery, patient stratification, and the development of targeted antifibrotic therapies, thereby advancing precision medicine strategies and transforming liver fibrosis from a static pathological entity into a dynamic, multicellular disease with actionable therapeutic opportunities.

## 2 Literature search and study selection

### 2.1 Literature search strategy

The scale of single-cell RNA sequencing has expanded rapidly, enabling the profiling of increasingly large numbers of individual cells (26). To inform this narrative review, a literature search was initiated on 31 December 2025 using Google Scholar, PubMed, and Web of Science to identify original single-cell transcriptomic studies of liver fibrosis in animal models and human populations. The search combined terms related to liver fibrosis (“liver fibrosis”, “cirrhosis”, and “hepatic fibrosis”) with terms related to single-cell technologies (“single-cell RNA sequencing”, “scRNA-seq”, and “single-cell transcriptomics”). The studies summarized in **Tables 1**, **2**, with the selection process presented in **Supplementary Figure S1**, were limited to those published on or before 31 December 2025. The reference list also includes recent publications from 2026 to provide up-to-date methodological, mechanistic, and translational context.

**Table 1:** Single-cell transcriptomic study of animal models of liver fibrosis and cirrhosis.

Author, country, publication year	Data originality	Species/gender/age or weight/case vs. control (number)	Comparison	Disease model (method,dose,frequency,duration)	Intervention (method,dose,frequency,duration)	Sampling time	Cell number	Sequencing details	Statistics package	Cluster type [marker genes]/ trend	Key cell subsets [marker genes]	Pseudotime trajectory analysis	Cell–cell communication analysis	Other major findings	Validation	Database number
Cheng,China, 2023 (42)	Original data	C57BL/6J mice/male/6-8wks	CCl_4_ mice vs.Control mice	CCl_4_ group: intraperitoneal injection, CCL_4_ (1 mL/kg, 1:4 corn oil), twice per week, 6wks	None	6wks after CCl_4_ injection	NPCs(15,357)	10x Genomics	R(v3.5)	HSCs [Col3a1, Col1a2, Col1a1] / Increase	HSC0 [Saa3, Slpi]		1.HSCs are a highly trophic and interconnected population in the hepatic microenvironment.	1.Sdc4 was highly upregulated on aHSCs, but barely expressed on resting HSCs, serving as a novel pan-activation marker for HSCs.	1.IF: locate biomarkers in liver tissue and HSCs	CRA007803
				Control group: intraperitoneal injection, equivalent amount of oil, twice per week, 6wks					Seurat(v2.3.2)		HSC1 [Rgs5, Colec11]				2.WB: verify interaction between HSCs and macrophages	
									Monocle		HSC2 [Ebf1, Pi16]					
									CellPhone DB		HSC3 [Tnc, Tagln]					
										Macrophages [Lgals3, C1qc] / Increase	Mac0 [Mmp 12, Stmn1]	1.Ly6Chigh monocytes sustain early inflammation through cytokine/chemokine release before maturing into Ly6Clow MDMs.	2.Fibrotic macrophages secrete GF, chemokines, and adhesion molecules to drive HSC proliferation, activation, and ECM remodeling.			
											Mac1 [S100a4, Thbs1]		3.Macrophage-derived Thbs1 binds αVβ3/α2ββ3 on HSCs to trigger PI3K/AKT/mTOR–mediated activation and fibrosis.			
											Mac2 [Fabp1, Hbb-bs]					
											Mac3 [Kdr, Ehd3]					
										Endothelial cells (ECs) [Clec4g, Gpihbp1] / Decrease						
										B cells [Cd79a, Ccr7] / Decrease						
										NK/T Cells [Ccl5, Nkg7] / Decrease						
Bai,China,2023 (43)	Original data	C57BL/6 mice/male/5wks/3 vs.3	TCS-200 mice vs.Control mice	TCS-200 group: intragastric administration, TCS (200 mg/kg, 40 mg/mL corn oil), once daily, 14 days	None	14 days after intragastric administration of TCS	TCS-200 group(38,368)	10x Genomics(v3.1)	GraphPad Prism (v8)	HSCs [Acta2, Col1a1] / Increase	qHSC [Lrat, Rgs5]	1.qHSCs diverged into aHSC1 (ECM/fibrosis‑enriched) and aHSC4 (chemotaxis‑enriched) subtypes.	1.aHSC1 was the most interactive subtype, indicating a central role in fibrosis.		1.IF: Localize fibrosis-related proteins to show their distribution in liver.	CRA005439
				Control group: intragastric administration, corn oil (5 mL/kg), once daily, 14 days			Control group(37,841)	Illumina HiseqXTEN	R		aHSC1 [Fmod]				2.WB: Detect fibrosis-related proteins to confirm TCS-induced upregulation.	
									Seurat(v4.0.3)		aHSC2 [Acta2, Col1a1]					
									monocle(v2.20.0)		aHSC3 [Ccl6]					
									CellPhone DB (v2.1.4)		aHSC4 [Ccr2]					
											aHSC5 [Ccl6]					
											aHSC6 [Thbs1]					
											aHSC_prof [Mki67, Mcm6]					
										Kupffer cells [C1qa, Csf1r] / Decrease	M1 [Cd86, Cd68]	2.LCM and Kupffer cells underwent M1‑to‑M2 reprogramming, with proliferative expansion driving M2‑skewed polarization.		1.Kupffer and LCM macrophages polarized toward the M2 phenotype, with upregulated pro-fibrotic genes (IL-6, IL-1β, TGF-β) and enhanced ECM-remodeling gene expression.		
										Liver capsular macrophages (LCMs) [S100a4, Itgax]	M2 [Tgfb2, Wnt5a]					
											Proliferation [Mki67, Stmn1]					
										Endothelial cells (ECs) [Pecam1, Clec4g] / Decrease	LVEC_central [Vwf, Wnt9b, Rspo3]	3.The shift of LSEC_pc/mid2 cells toward LVEC_central (capillarization), driven by Hlf, is associated with liver fibrosis.		2.LSEC_mid2 subset expanded significantly, with upregulated ECM remodeling and TGF-β signaling, and impaired endocytosis (downregulated Mrc1, Stab1/2).		
											LVEC_portal [Gja5, Ntn4]					
											LSEC_pc [Kit]					
											LSEC_mid [Fcgr2b, Clec4g]					
											LSEC_mid2 [Hamp]					
											LSEC_mid/pp [Lyve1]					
											LSEC_pp [Gpr182]					
										B cells [Cd79a, Cd79b, Ms4a1] / Decrease	B_naive [Ighd, Fcmr]	4.B_naive cells adopted two major fates: B_plasma and B_memory, the latter co‑occurring with a minor B_plasma population.				
											B_memory [Cd38]					
											B_plasma [Igha, Jchain]					
										T/NK cells [Nkg7] / Decrease	CD4_Treg [Foxp3, Ctla4]					
											CD4_naive [Lef1]					
											CD4_Tem [Cd40lg]					
											CD8_naive [Cd8b1]					
											CD8_CTL [Gzmk]					
											NKT [Cd3g, Xcl1]					
											NK_cyto [Prf1]					
											NK_inflam [Xcl1]					
Yang,China,2023 (62)	Original data	ICR mice/30.0±1.0g/6wks/6 vs.6	CCl_4_+si Plgrkt mice vs.Control+si Plgrkt mice	CCl_4_+si Plgrkt group: intraperitoneal injection, CCL_4_ (1 μL/g, 1:9 corn oil), twice per week, 4wks	Intraperitoneal injection, Plgrkt siRNA-GeRPs (1.5 mg GeRPs/kg), the day after CCl_4_ injection, 4wks	1, 2, 7, and 28 days post-CCl_4_ injection	CCl_4_ group(3,750)	10x Genomics	GraphPad Prism(v9)	Macrophages / Increase	BMM-1	1.BMMs converted to SAMs during fibrosis, losing monocyte markers and acquiring SAM signature genes.	1.Highly expressed in SAMs, Plg‑RKT—together with PLG—plays a key role in BMM‑to‑SAM transformation.		1.IF: Localize macrophages and siRNA-GeRPs to verify specific uptake.	CRA007105
				CCl_4_+si Nc group: intraperitoneal injection, CCL_4_ (1 μL/g, 1:9 corn oil), twice per week, 4wks	Intraperitoneal injection, Nc siRNA-GeRPs (1.5 mg GeRPs/kg), the day after CCl_4_ injection, 4wks		BDL group(11,634)	Illumina HiSeq X Ten	Monocle2		SAMs(BMM-2/8) [Cd9, Trem2, Lgals3]	2.BMM‑to‑SAM differentiation was markedly enhanced in fibrotic livers but minimal in normal livers.	2.Plg-RKT-PLG promotes the transformation of SAMs in liver fibrosis.		2.WB: Detect FN1, LOXL2, COL1A1 to confirm profibrotic protein changes.	
				Control+si Plgrkt group: intraperitoneal injection, equivalent amount of oil, twice per week, 4wks	Intraperitoneal injection, Plgrkt siRNA-GeRPs (1.5 mg GeRPs/kg), the day after CCl_4_ injection, 4wks		Control group(8,195)		CellChat		BMM-3				3.RNA-seq: Identify SAMs, their origin and Plg-RKT expression in liver NPCs.	
				Control+si Nc group: intraperitoneal injection, equivalent amount of oil, twice per week, 4wks	Intraperitoneal injection, Nc siRNA-GeRPs (1.5 mg GeRPs/kg), the day after CCl_4_ injection, 4wks						BMM-4					
		ICR mice/30.0±1.0g/6wks/6 vs.6	BDL+si Plgrkt mice vs.Sham+si Plgrkt mice	BDL+si Plgrkt group: BDL	Intraperitoneal injection, Plgrkt siRNA-GeRPs (1.5 mg GeRPs/kg), twice per day, 2wks	1, 3, 7, and 14 days post-bile duct ligation					BMM-5					
				BDL+si Nc group: BDL	Intraperitoneal injection, Nc siRNA-GeRPs (1.5 mg GeRPs/kg), twice per day, 2wks						BMM-6/7					
				Sham+si Plgrkt group: Sham surgery	Intraperitoneal injection, Plgrkt siRNA-GeRPs (1.5 mg GeRPs/kg, twice per day, 2wks						Kupffer cell					
				Sham+si Nc group: Sham surgery	Intraperitoneal injection, Nc siRNA-GeRPs (1.5 mg GeRPs/kg), twice per day, 2wks											
Yang,China,2021 (31)	Original data	C57BL/6J mice/male/8-12wks/3 vs.3	CCl_4_ mice vs.Control mice	CCl_4_ group: intraperitoneal injection, CCL_4_ (1 mL/kg, 1:5 corn oil), twice per week, 3wks	None	24 hours after the final injection	CCl_4_ group(18,185)	10x Genomics	GraphPad Prism(v8)	HSCs [Colec11, Ecm1] / Increase	qHSC [Lrat, Ecm1]	1.CCL_4_-induced HSC activation involved ordered remodeling of gene modules: from inflammation to migration/contraction, then to ECM synthesis.	NR	1.In the CCl_4_ model, the activated HSCs are mainly stage-2 and stage-3.	1.bulk RNA-seq: Analyze gene expression in HSCs to validate activation pathways and sGC regulator effects.	1.GSE171904
				Control group: intraperitoneal injection, equivalent amount of oil, twice per week, 3wks			BDL group(18,931)	Illumina Novaseq	R		aHSC(Stage-1) [Ccl2, Cxcl10]			2.In the BDL model the activated HSCs are mainly stage-1.	2.qPCR: Quantify mRNA levels of HSC/PF markers and fibrosis-related genes.	2.GSE171885
		C57BL/6J mice/male/8-12wks/3 vs.3	BDL mice vs.Sham mice	BDL group: BDL		10 days post-bile duct ligation	Control group(10,636)		Seurat(v3)		aHSC(Stage-2) [Acta2, Tpm1]				3.IF: Localize and identify HSCs, PFs, collagen, and activation markers in liver tissue.	
				Sham group: Sham surgery					Slingshot		aHSC(Stage-3) [Col1a1, Col1a2, Col3a1]					
Su,United States,2021 (76)	Original data	Cdh5-CreERT2, mT/mG mice/male/4wks	cirrhotic mice vs.control mice	CCl_4_ group: inhalation, vaporized CCl_4_, 12wks	Intraperitoneal injection, 100 mg/g tamoxifen, once per day, 5 days	12wks after CCl_4_ inhalation	Cirrhosis group(4,076)	10x Genomics	GraphPad Prism(v7)	Endothelial cells (ECs) / Decrease	Arterial-like ECs (EC1) [Gja5, Vwf]	NR	NR	1. Capillarization in LSECs was most prominent in zone 3, characterized by CD34 induction in cirrhotic livers.	1.IF: Localize EC subtypes, capillarization and EndMT markers in liver tissue.	GSE147581
				Control group: inhalation, clean air, 12wks	Intraperitoneal injection, 100 mg/g tamoxifen, once per day, 5 days		Control group(3,248)	Illumina HiSeq 4000	Seurat(v3.0)		LSECs(EC2),periportal (zone 1) [Msr1, Ntn4]			2. In cirrhotic mice, LSECs showed marked downregulation of endocytic receptors (Mrc1, Stab1/2, Scarb1/2) and Lamp2, particularly in zone 3.	2.WB: Detect LSEC and EndMT protein expression in cultured LSECs.	
		Cdh5-CreERT2, mT/mG mice/male/2mos		BDL group: BDL	Intraperitoneal injection, 100 mg/g tamoxifen, once per day, 5 days	1, 2, and 4 wks post-bile duct ligation			ClusterProfiler		LSECs (EC3), midzonal (zone 2) [v]			3. Expression of shear stress–responsive transcription factors (Klf2, Klf4, AP-1) in LSECs was downregulated in cirrhotic mice, likely contributing to increased intrahepatic vascular resistance.	3.qPCR: Quantify LyEC-specific genes in human primary LSECs and LyECs.	
				Sham group: Sham surgery	Intraperitoneal injection, 100 mg/g tamoxifen, once per day, 5 days						LSECs (EC4), pericentral(zone 3) [Wnt2, Kit					
											Central venous ECs (EC5) [Wnt9b, Lhx6]					
											Lympatic EC6 [Meox1, Tbx1]					
Zhang,United States,2021 (44)	Original data	C57BL/6 mice/male/8wks	CCl_4_ mice vs.Control mice	CCl_4_ group: intraperitoneal injection, CCL_4_ (2 mL/kg, 1:1 corn oil), three per week, 2wks	None	2wks after CCl_4_ injection	HSC1(331)	10× Genomics	Seurat(v3.1.4)	HSCs [Fgl2, Rgs5]	HSC1	1.HSC1 served as the root, giving rise to intermediate subpopulations (HSC2-4) and terminal subpopulations (HSC5-6).		1.HSC subpopulations segregate spatially: PV HSCs mainly differentiate into HSC4/HSC3, while CV HSCs generate HSC1, HSC2, and HSC3.	1.IF: Identify HSC subtypes and verify marker co-localization in mouse and human fibrotic liver.	NR
		LRAT-Cre:Rosa26mT/mG mice		Control group: intraperitoneal injection, equivalent amount of oil, three per week, 2wks			HSC2(1,375)	Illumina NextSeq500	monocle 3(v5)		HSC2	2.HSC2, the largest subpopulation, is marked by the expression of myofibroblast markers such as α-SMA.			2.IHC: Detect Rgs5 expression and localize HSCs in human cirrhotic liver tissue.	
							HSC3(1,146)		CellRanger(v3.1.0)		HSC3	3.HSC3 is distinguished by the expression of chemokines involved in inflammation.	1.HSC3 orchestrates inflammation and ECM deposition in liver remodeling under injury.			
							HSC4(974)				HSC4		2.HSC4 is enriched in genes for tissue repair, vasoactive response, and angiogenesis.			
							HSC5(321)				HSC5					
							HSC6(302)				HSC6 [mt-Co3, mt-Atp6]					
							HSC7(39)				HSC7					
							HSC8(109)				HSC8					

B cells, B lymphocytes; BDL, Bile Duct Ligation; BBM, Bone Marrow-derived Monocytes/Macrophages; CHB, chronic hepatitis B; ECs, Endothelial cells; HBV, hepatitis B virus; HC, Healthy Control; HSCs, Hepatic stellate cells; IF, Immunofluorescence; IHC, Immunohistochemistry; LF, Liver Fibrosis; MFBs, Myofibroblasts; MP, Mononuclear Phagocytes; NK cells, Natural Killer cells; NR, not reported; qRT, quantitative real-time; SAMs, scar-associated macrophages; WB, western blot; T cells, T lymphocytes; wks, weeks.

**Table 2:** Single-cell transcriptomic study of human liver fibrosis and cirrhosis.

Author, country, publication year	Gender(male)/ age or weight	Comparison (number)	Sampling	Biochemical indicators	Cell number	Sequencing details	Statistics package	Cluster type [marker genes]/ trend	Key cell subsets[marker genes]	Pseudotime trajectory analysis	Cell–cell communication analysis	Other major findings	Validation	Database number
Zhuo,China,2025 (67)	female:male=1:4/43.6±17.91	HBV(5) vs. HC(3)	Livers	ALT: 181.12±313.19U/L	Monocyte(9,593)	10x Genomics	GraphPad Prism (v10.1.2)	Monocytes [CD14^+^, CD16^+^] / Increase	C1_CD14_S100A12	1. The fate is mainly from non-classical monocytes, intermediate monocytes, and with classical monocytes as the end.	NR	1. The MPA subcluster cells secrete significantly higher levels of CCL5 and PDGF-A compared to non-MPA cells, and these factors play important roles in liver fibrosis.	1.IF: Verify LMO2 expression and localization in human monocytes.	CNP0006627
				AST: 141.5±488.95U/L		Illumina sequencing platform	R(v4.0.3)		C2_CD14_CCL4			2.MPA induces proinflammatory cytokine production in LX-2 cells via CCL5 secretion, thereby exacerbating liver inflammation and fibrosis.	2.WB: Confirm LMO2 overexpression in THP-1 cells.	
	female:male=2:1/27.67±2.52						Seurat		C3_CD14_LGALS2					
							Monocle 3		C4_CD14_ITGA2B					
									C5_CD14_FCGR3A (CD16)					
									C6_FCGR3A (CD16)					
					T cell(21,486)			T cells [CD3D^+^]						
					NKT(5,524)			NKT cells [CD3D^+^, NKG7^+^]						
					NK(4,131)			NK cells [CD3D^-^, NCR1^+^]						
					B cell(4,575)			B cells [CD79A]						
Bai,China,2023 (63)	female:male=1:1/51.0±5.7	HBV(5) vs. HC(6)	Livers	ALT: 181.12±313.19U/L	Immune cells(45,993)	10x Genomics	Seurat(v3.2)	Myeloid cells [S100A9, IFI30, LYZ, MS4A6A, CST3, CTSS, AIF1]	Monocyte-S100A6 [S100A6, S100A9]	1.Single-cell trajectory analysis in HBV cirrhosis showed a shift from monocytes to mature macrophage/dendritic cells, indicating evolution toward a pro-fibrotic/pro-inflammatory immune microenvironment.	1.Myeloid cells increased interaction with all immune cells in cirrhosis, primarily via the CD74/CXCR4 pathway.	1.Antigen-processing and MHC-II presentation capacities are significantly up-regulated, while ROS-response and MAPK signalling are down-regulated.	1.IF: Validates the upregulation and localization of C1QA, CD9, IL18 in HBV cirrhotic liver tissues.	GSE230022
	female:male=0:2/41.0±31.1			AST: 141.5±488.95U/L		MGISEQ-2000 sequencing platform	monocle(v2.22.0)		Monocyte-S100A9 [S100A6, S100A9]					
	female:male=0:3/61.7±7.5						CellChat(v1.6.1)		Monocyte-FCGR3A [FCGR3A]					
	NR								Macrophage-C1QA [C1QA]					
									Macrophage-CCL5 [CCL5]					
									Macrophage-CD9[CD9]/IFI6					
									Macrophage-CD9/IL18 [IL18, CD9]					
									Macrophage-NAMPT [NAMPT]					
									Macrophage-PLCG2 [PLCG2]					
									Macrophage-THBS1 [THBS1]					
									pDC [GZMB]					
									cDC1 [SLAMF7]					
									cDC2 [CD1C]					
								T cells [IL7R, IL32]						
								B cells [MS4A1, CD79A, CD79B]				2.Naïve B cells almost disappear; the activation-linked B-cell-IGLC1 subset is significantly reduced.		
								NK cells [GNLY, NKG7]						
Ai,China,2022 (45)	NR	LF(9) vs. HC(11)	Livers	NR	62,210cells	10x Genomics	Seurat (v4.0.5)	HSCs [RGS5, ACTA2]	NR	1.During liver injury, hepatic stellate cells transdifferentiate into proliferative, fibrotic myofibroblasts (MFBs).	1.Key molecules mediating communication between hepatic stellate cells (HSCs) and myofibroblasts include ALX, PDGFA, MDK, AGT, PGF, NTF3, and JAG1 from HSCs, and GAS6, PDGFRA, LRP1, PROS1, and HGF from myofibroblasts.	1.Immune system activation drives stellate cell activation in cirrhosis by releasing inflammatory factors.	1.IHC: Validates APOE upregulation in human cirrhotic liver.	GSE136103
							monocle3(v1.0)	Myofibroblasts(MFBs) [RBP1, COL1A1]				2.THY1, MMP2, COL3A1, PCOLCE, SERPINF1, COL1A2, and COL1A1 are strongly associated with APOE expression, with several serving as key driver genes in myofibroblasts.		
							CellPhoneDB(v2.1.7)	NKT cells [CD3D, CD3G]						
Ramachandran,UK,2019 (23)	female:male=4:1/56.6±5.8	LC(5) vs.HC(5)	livers	NR	66,135 cells	10x Genomics	Seurat v2.3.0	Mononuclear Phagocytes(MP) [FCN1, VCAN]	MP(1) TM(1) [S100A12, FCN1]		1.Extensive ligand–receptor interactions occur among the expanded pathogenic cells (SAMs, SAEndos, SAMes) in cirrhosis.	1.Cirrhosis remodels non-parenchymal cells, with SAMs, SAEndos, and SAMes markedly expanded.	1.IF: Localize and verify TREM2, CD9, PLVAP, PDGFRα in fibrotic liver niche.	GSE136103
	female:male=3:2/57.4±7.9					Illumina HiSeq 4000	Monocle(v.2.6.1)		MP(2) TM(2) [NBEAL1]				2.IHC: Quantify TREM2, CD9, TIMD4, MARCO expression in healthy and cirrhotic livers.	
							CellPhoneDB		MP(3) TM(3) [IFITM2, APOBEC3A]					
									MP(4) SAM(1) [RGS2]	1.In the fibrotic liver, the abundantly expanded TREM2^+^CD9^+^ scar-associated macrophages (SAMs) are derived from monocytes.	2.SAMs activate PDGFR and TNFRSF12A signaling to promote SAMes proliferation and expansion.			
									MP(5) SAM(2) [TREM2, CD9]					
									MP(6) KC(1) [CD5L, CD163]					
									MP(7) KC(2) [ARL4C]					
									MP(8) cDC2 [FCER1A, CD1E]					
									MP(9) cDC1 [LGALS2]					
									Cycling					
								Endothelias [CLEC14A, PLVAP]	Endo(1) [CLEC4G, LYVE1]					
									Endo(2) [CCL21, PROX1]					
									Endo(3) [CPE, CD320]					
									Endo(4) [RSPO3, WNT2]					
									Endo(5) [AIF1L, SOX17]					
									Endo(6) [PLVA, VWA1]					
									Endo(7) [ACKR1]					
								Mesenchymes [RGS5, ACTA2]	Mes(1) VSMC [MYH11]					
									Mes(2) HSC [RGS5]					
									Mes(3) SAMes [COL3A1, COL1A1]	2.PDGFRα^+^ SAMes, the key fibrogenic cells in scar formation, originate from quiescent HSC differentiation.	3.SAEndos express Notch ligands that engage NOTCH3 on SAMes to induce collagen synthesis.	2.SAMs show a profibrotic phenotype, secreting mediators that induce collagen in HSCs.		
									Mes(4) Mesothelia [KRT19, PDPN, WT1, MSLN]					
								T cells [MKI67, TOP2A]						
								В сеlls [CD79A, MS4A1]						

B cells, B lymphocytes; CHB, chronic hepatitis B; HBV, hepatitis B virus; HC, Healthy Control; HSCs: Hepatic stellate cells; IF, Immunofluorescence; IHC, Immunohistochemistry; LF, Liver Fibrosis; MFBs, Myofibroblasts; MP, Mononuclear Phagocytes; NK cells, Natural Killer cells; NR, not reported; SAMs, scar-associated macrophages; SAMes, scar-associated mesenchymal cells; SAEndos, scar-associated endothelial cells; WB, western blot; T cells, T lymphocytes.

### 2.2 Study selection and data extraction

The initial search identified 10,881 records: 10,377 from Google Scholar, 303 from PubMed, and 201 from Web of Science. Records from the three sources were merged in EndNote, and 468 duplicates were removed, leaving 10,413 records. Restricting the results to original research articles excluded 3,316 non-research records and retained 7,097 records. Title and abstract screening excluded a further 6,984 records, leaving 113 reports for eligibility assessment.

Articles were included if they investigated liver fibrosis using single-cell transcriptomics and were mainly based on comparative data from fibrotic and normal liver conditions in animal models or human populations. Of the 113 reports assessed, 103 did not meet these combined criteria. The final synthesis included 10 original research articles: six animal studies and four human studies. The study selection process is summarized in **Supplementary Figure S1**, using a flow diagram adapted from PRISMA 2020 (27).

For each included article, information on species, disease etiology/model, sample source, sequencing platform, analytical approaches, and major findings was extracted. Due to differences in experimental designs and computational pipelines, findings were qualitatively synthesized rather than quantitatively pooled. No meta-analysis was performed.

### 2.3 Cross-study comparison criteria

Cluster marker genes were defined as genes enriched in specific cell populations reported by the original studies, whereas DEGs referred to statistically identified changes between disease and control conditions. Pathway findings were compared based on reported biological themes rather than identical gene lists, considering differences in species, disease models, and analytical methods.

## 3 Cellular heterogeneity in liver fibrosis revealed by single-cell transcriptomics

Single-cell transcriptomics has transformed the study of liver fibrosis by resolving cellular heterogeneity in human tissues and experimental models (23, 28). Across carbon tetrachloride (CCl_4_) injury, bile duct ligation, diet-induced steatohepatitis, and genetic fibrosis models, sc/snRNA-seq identifies fibrogenic mesenchymal cells, activated macrophages, endothelial remodeling states, and lymphoid changes across disease stages (29–31). Human studies complement these findings by revealing disease-stage–specific states, including scar-associated macrophages, heterogeneous stellate cell subsets, and capillarized endothelial phenotypes (32). Cross-species analyses further support integrated mapping of cellular states and communication networks (33, 34).

To summarize these advances, we integrated findings from published animal models (**Table 1**) and human cohorts (**Table 2**). Because most studies focus on specific cell populations, findings across cell types reflect different experimental contexts. Human and animal studies are therefore discussed separately, with human studies including HBV-related disease and mixed-etiology cirrhosis and animal studies including CCl_4_, BDL, and TCS models. The following sections focus on cellular heterogeneity, recurrent molecular programs, and intercellular communication in major fibrogenic and immune populations.

### 3.1 Hepatic stellate cells

HSCs are the principal fibrogenic mesenchymal cells in the liver and a major source of extracellular matrix during chronic injury, with increasing abundance and activation reported as fibrosis progresses (**Supplementary Table S1**) (35, 36). Under inflammatory, toxic, or metabolic stress, quiescent vitamin A–storing HSCs acquire proliferative and contractile myofibroblast-like features and increase collagen production (37), accompanied by marked changes in collagen, ECM-remodeling, and fibrogenic genes (38). Single-cell profiling further resolves heterogeneous activation and transitional states that are not evident in bulk analyses (39, 40) and reveals potential molecular pathways and interactions between HSCs and immune or endothelial cells (41).

Murine single-cell studies have identified partially overlapping HSC programs across different fibrosis models. Zhang et al. and Cheng et al. studied CCl_4_-induced fibrosis in C57BL/6J mice, Yang et al. examined BDL- and CCl_4_-induced fibrosis, and Bai et al. used a triclosan (TCS)-induced model (31, 42–44). Despite these different injury contexts, Col1a1, Col1a2, and Col3a1 were repeatedly reported, reflecting shared transcriptional features related to collagen synthesis and ECM remodeling.

Comparison with the human cohort analyzed by Ai et al. further identified shared HSC-associated markers, including RGS5 and ACTA2 (45). ACTA2 marks contractile myofibroblast-like cells, whereas RGS5 is associated with cell migration and vascular-related mesenchymal phenotypes (46, 47).

A Venn diagram analysis (**Figure 1A**) comparing reported pathway annotations across four studies identifies three frequently enriched pathways: ECM organization, PI3K–Akt signaling, and ECM–receptor interaction (48). These pathways are closely linked to HSC activation and matrix deposition (49), with functional evidence supporting a role for PI3K–Akt signaling (50, 51). Cai et al. showed that insulin–PI3K–Akt–p70S6K activation promotes HSC activation in experimental steatohepatitis (52), whereas Son et al. found that PI3K inhibition reduces HSC proliferation, collagen synthesis, and ECM deposition *in vivo* (50).

**Figure 1:**
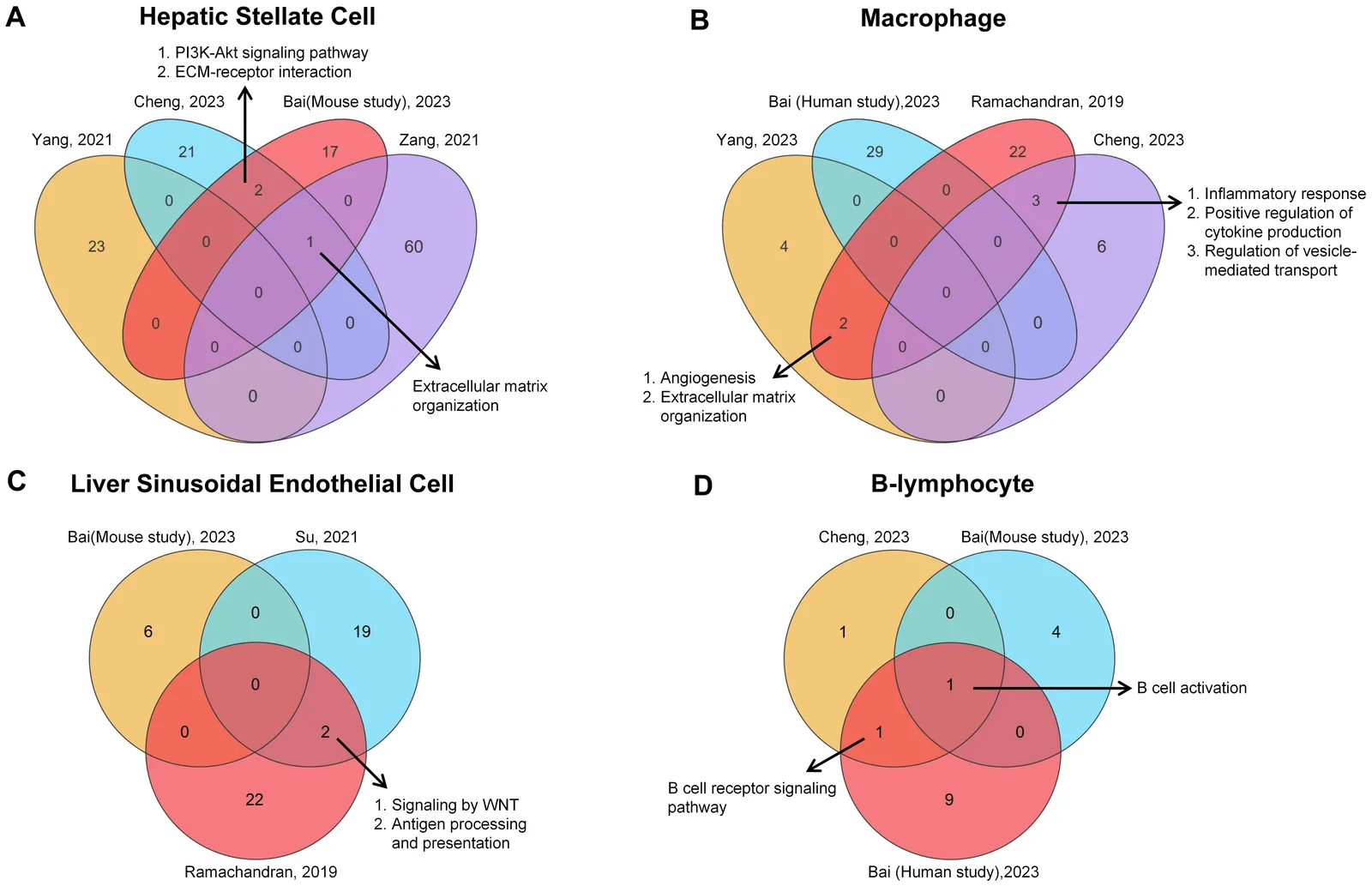
Venn diagrams of enriched pathways in key hepatic cell types during liver fibrosis. **(A)** Venn diagram of enriched pathways in Hepatic Stellate Cells (HSCs). **(B)** Venn diagram of enriched pathways in Macrophages. **(C)** Venn diagram of enriched pathways in Liver Sinusoidal Endothelial Cells (LSECs). **(D)** Venn diagram of enriched pathways in B lymphocyte. These overlaps reflect shared biological themes rather than conserved mechanisms. Differences in species, disease models, and analytical approaches limit direct pathway comparisons and preclude conclusions regarding fibrosis-specific regulation.

ECM organization and ECM–receptor interaction reflect extensive matrix remodeling during fibrosis. Activated HSCs produce collagen I and fibronectin and respond to integrin-mediated mechanotransduction from the stiffened matrix, further reinforcing activation. These findings are consistent with single-cell results implicating PI3K–Akt and ECM–integrin signaling in HSC activation and matrix accumulation (53).

Beyond these established pathways, the major contribution of single-cell transcriptomics is its ability to resolve functional HSC heterogeneity. Distinct HSC subsets can show inflammatory, pro-tumorigenic, regenerative, or fibrosis-resolving features. Filliol et al. demonstrated opposing roles of HSC subpopulations in hepatocarcinogenesis (54), while other studies highlight the context-dependent functions and therapeutic implications of HSC heterogeneity (35, 55). Thus, single-cell studies extend conventional activation models by linking specific HSC states to distinct disease-related programs.

### 3.2 Kupffer cells and macrophages

Macrophages are key regulators of inflammation, immune responses, and fibrogenic niche formation in chronic liver injury (56, 57). Single-cell studies reveal marked macrophage heterogeneity and diverse activation states within fibrotic livers (58), involving both resident Kupffer cells and infiltrating monocyte-derived macrophages (59). Across experimental systems, increased hepatic macrophage abundance has also been associated with fibrosis severity (**Supplementary Table S1**) (60, 61).

Murine studies using CCl_4_- and TCS-induced fibrosis have identified partially overlapping macrophage signatures (42, 43, 62). Recurrently reported genes include Lgals3, Stmn1, and S100a4, which are associated with activation, migration, cytoskeletal remodeling, and fibrogenic signaling. These similarities suggest shared macrophage features across injury models rather than a uniform transcriptional program.

Human single-cell studies further support the relevance of fibrosis-associated macrophage states. In HBV-related and mixed-etiology cirrhosis, respectively, Bai et al. and Ramachandran et al. identified CD9 as a shared macrophage marker (23, 63). Animal–human comparisons also revealed partially shared signatures, including CD9, TREM2, THBS1, C1QA, and C1QC, although their interpretation may vary by species and etiology. These populations resemble scar-associated or lipid-associated macrophages linked to ECM remodeling and stromal–immune crosstalk (21, 34).

A Venn analysis of four representative studies (**Figure 1B**) identified recurrent enrichment of inflammatory response, ECM organization, angiogenesis, cytokine production, and vesicle-mediated transport (64). Functional studies support these themes: Karlmark et al. showed that recruited Ly6C^+^ monocytes promote fibrosis through profibrogenic macrophage differentiation (65), whereas Krenkel et al. found that CCR2/CCR5 blockade reduces inflammation and fibrosis in experimental NASH (66). In patients with chronic HBV infection, Zhang et al. identified monocyte subsets associated with fibrosis severity (67). Together, these findings support macrophages as candidate targets for antifibrotic intervention (68).

### 3.3 Liver sinusoidal endothelial cells

LSECs maintain hepatic vascular homeostasis, immune surveillance, metabolic exchange, and tissue repair (69, 70). During fibrosis, single-cell studies reveal marked changes in endothelial identity, zonation, and signaling (71, 72). LSEC abundance has been reported to decline with fibrosis progression (**Supplementary Table S1**) (73), alongside capillarization, loss of fenestration, and impaired homeostatic function. These changes have been linked to pathways such as YAP–GATA6–eNOS, contributing to microvascular dysfunction and a fibrogenic microenvironment (74, 75).

Murine single-cell studies have identified shared endothelial features across CCl_4_-, BDL-, and TCS-induced fibrosis. Su et al. examined CCl_4_ and BDL models, whereas Bai et al. used TCS-induced injury (43, 76). Recurrently reported markers included Vwf, Wnt9b, Gja5, Kit, Ntn4, and Lyve1, reflecting endothelial subpopulation and signaling features during fibrotic remodeling.

Human studies show similar LSEC-associated transcriptional features. In mixed-etiology cirrhosis, Ramachandran et al. identified endothelial markers including CLEC4G, LYVE1, and RSPO3 (23), supporting similarities between experimental models and human fibrotic disease.

A Venn analysis of three representative studies (**Figure 1C**) identified recurrent enrichment of antigen processing and presentation and WNT signaling (77). Functional evidence supports important roles for LSEC capillarization and immune regulation: DeLeve et al. showed that endothelial dedifferentiation precedes HSC activation and promotes fibrogenesis (78), while Poisson et al. described LSECs as active regulators of antigen presentation and cytokine signaling (69). Together, these findings highlight endothelial capillarization and WNT-related signaling in fibrotic vascular remodeling and suggest potential biomarker and therapeutic relevance (79, 80).

### 3.4 B lymphocytes

B lymphocytes support hepatic immune surveillance through antibody production, antigen presentation, and cytokine secretion. Single-cell studies indicate changes in B-cell abundance and functional states during fibrosis (81, 82). Although intrahepatic B-cell proportions may remain relatively stable, modest increases in absolute numbers have been reported in CCl_4_ models and human fibrotic liver (**Supplementary Table S1**) (83), potentially reflecting enhanced recruitment and local activation (84).

Across murine and human studies, B-cell populations share several transcriptional features. Cheng et al. used CCl_4_-induced fibrosis and Bai et al. used a TCS-induced model (42, 43), while human studies of HBV-related cirrhosis identified the canonical B-cell markers MS4A1, CD79A, and CD79B (23, 63, 67). These genes support consistent B-cell identity and receptor-related signaling across datasets.

A Venn analysis integrating two animal studies and one human study (**Figure 1D**) identified recurrent B-cell programs across models and HBV-related cirrhosis (42, 43, 63). B-cell activation and B-cell receptor (BCR) signaling were repeatedly enriched, with B-cell activation showing the broadest overlap (81). Functional studies further support a role for B cells in fibrosis: B-cell-deficient mice show reduced fibrosis (85), and mesenchymal stem cells can attenuate fibrosis by suppressing pathogenic intrahepatic B-cell activity (81). These findings suggest that B-cell activation and BCR-related signaling are common features of immune remodeling and may have biomarker or therapeutic relevance (86).

### 3.5 Integrated pathway analysis across major cell types in liver fibrosis

Liver fibrosis emerges from coordinated interactions among mesenchymal, immune, and endothelial compartments rather than isolated cellular programs (41). HSCs, macrophages, LSECs, and lymphoid cells interact through signaling pathways involved in inflammation, ECM remodeling, vascular dysfunction, and immune regulation (87). Integrating these pathway-level findings can therefore help identify shared biological processes and potential therapeutic nodes (88).

Across studies, antigen processing and presentation, response to wounding, and ECM organization were recurrently reported in HSCs, macrophages, and LSECs (23, 42–44, 62, 76). Antigen presentation extends beyond classical immune cells to stromal and endothelial populations (89, 90), while wound-response and ECM programs reflect coordinated injury sensing, matrix deposition, and vascular adaptation during fibrosis (91).

Pairwise comparisons also revealed cell type–specific pathway overlap. HSCs and macrophages shared enrichment of the viral myocarditis pathway, largely reflecting inflammatory modules involving cytokines, stress responses, and immune activation (42, 63). Components of this pathway, including interferon and inflammatory signaling, are also implicated in chronic liver injury and fibrosis (92, 93).

HSCs and LSECs shared ECM–receptor interaction and platelet-related pathways, linking matrix remodeling with vascular dysfunction (42–44, 76). ECM–receptor signaling contributes to mechanotransduction and HSC activation, while platelet signaling can influence endothelial activation, microvascular remodeling, and profibrotic cytokine release (94–96). Together, these pathways highlight a matrix–vascular interface in fibrotic remodeling.

Macrophages and B lymphocytes shared enrichment of the phagosome pathway, reflecting overlap in antigen uptake, processing, and immune regulation (23, 63). This may indicate shared antigen-processing functions beyond their conventional immune roles (97, 98) and illustrates the functional plasticity of immune cells in the fibrotic liver (99).

These findings are summarized in **Figure 2**, which integrates HSCs, macrophages, LSECs, and B lymphocytes with their shared and cell type–specific pathways into a unified framework of liver fibrosis. This integrated pathway landscape also has potential clinical relevance. Recurrent pathways such as antigen processing and presentation, ECM–receptor interaction, and phagosome-related signaling may represent shared regulatory nodes (100). Targeting such pathways could potentially affect multiple pathogenic cell populations and support antifibrotic therapy, biomarker development, and pathway-based patient stratification (101, 102).

**Figure 2:**
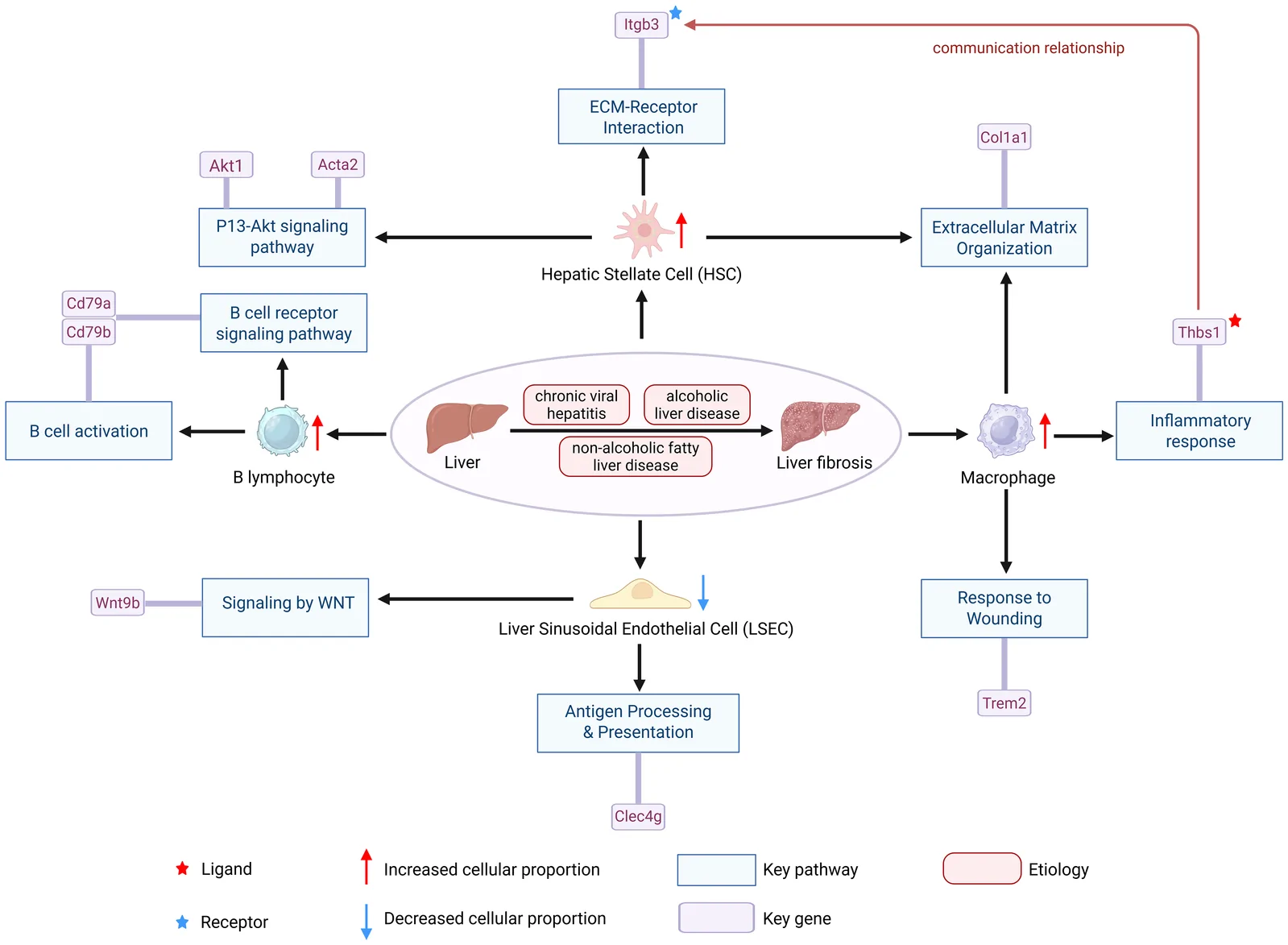
Conceptual overview of cell states, pathways, and intercellular interactions in liver fibrosis. Blue boxes indicate reported pathways, and purple boxes represent representative genes. Red and blue stars denote ligands and receptors, respectively. This schematic summarizes findings from diverse human and animal studies. Cell proportion changes may vary across species and disease models. This figure was created with BioRender.

### 3.6 Lobular zonation in liver fibrosis

Lobular zonation provides spatial context for hepatic cell states. Halpern et al. in the Itzkovitz laboratory combined scRNA-seq with spatial landmark genes to reconstruct hepatocyte positions along the lobule (103). Dobie et al. subsequently identified portal- and central-vein-associated HSC populations and showed that central-vein-associated HSCs were major collagen-producing cells in centrilobular CCl_4_ injury (104). This finding links HSC location to injury response but should not be generalized to all etiologies.

Zonation analysis has since been extended to human fibrosis using spatial transcriptomics and integrated single-nucleus profiling (28, 33). However, dissociation removes positional information, HSC activation reduces distinguishing zonation-associated expression (33), and fibrotic septa and regenerative nodules distort normal lobular architecture. Mapping diseased cells onto a healthy reference can therefore be misleading. Combining expression-based reconstruction with tissue imaging and spatial measurements is needed to distinguish original location from injury-induced state changes. These constraints help explain why zonation reconstruction has not become routine in every fibrosis study.

## 4 Single-cell trajectory analysis reveals dynamic cellular state transitions during liver fibrogenesis

Liver fibrosis is a dynamic and progressive process characterized by continuous transitions of multiple hepatic cell populations rather than static cell states (105). Traditional bulk or cluster-based single-cell analyses capture transcriptional heterogeneity but fail to reconstruct the temporal ordering of cellular activation, differentiation, and fate commitment that underlies fibrogenesis (106). Cellular trajectory inference, commonly implemented as pseudotime analysis in single-cell RNA sequencing (scRNA-seq) studies, enables reconstruction of continuous cell-state transitions along fibrotic progression, providing mechanistic insights into how quiescent or homeostatic cells evolve into pathogenic effector populations (107).

In the context of liver fibrosis, trajectory analysis has proven particularly powerful in identifying activation hierarchies, branch points, and terminal fibrogenic states across HSCs, macrophages, LSECs, and lymphoid populations (108). Importantly, related transcriptional programs have been reported between experimental mouse models and human fibrotic livers, highlighting shared regulatory programs underlying fibrogenesis (28, 109). From a translational perspective, trajectory-based frameworks allow identification of early transitional states, reversible activation nodes, and pathway-level regulators, offering opportunities for therapeutic intervention, biomarker development, and patient stratification beyond single marker or single cell-type approaches (110). Selected pseudotime-inferred cell-state trajectories and their associated molecular features identified in recent single-cell studies are summarized in **Supplementary Table S2**.

### 4.1 Hepatic stellate cell trajectories: from quiescence to fibrogenic effector states

HSCs are the dominant fibrogenic effector population in chronic liver disease, and trajectory-based single-cell transcriptomic analyses have substantially reshaped current understanding of their activation dynamics. Rather than representing a binary transition from quiescent to activated states, accumulating evidence from pseudotime and lineage inference analyses demonstrates that HSC activation follows a continuous, temporally ordered process, during which cells progressively acquire inflammatory, contractile, migratory, and extracellular matrix (ECM)–producing functions (31). These advances mark a shift from static cell-state descriptions toward a dynamic, process-oriented framework for HSC biology in liver fibrosis (111).

In experimental models of liver fibrosis, early trajectory analyses provided compelling evidence for a largely linear activation continuum. Using CCl_4_-induced fibrosis, Yang et al. inferred a continuous transcriptional continuum among HSC states, revealing that inflammatory programs are engaged early, followed by the emergence of migratory and contractile features, and ultimately associated with transcriptional states characterized by fibrogenic features characterized by robust ECM production (43). This stepwise ordering supports the classical concept of progressive HSC activation, while offering molecular resolution to previously inferred histopathological stages. Importantly, the temporal structure identified by pseudotime analysis suggests that inflammatory activation is not merely a byproduct of fibrosis, but an integral and early component of HSC fate commitment.

Subsequent work has expanded this linear model by revealing increased complexity and heterogeneity at later stages of HSC activation. In an independent CCl_4_-based study, Zhang et al. demonstrated that activated HSCs do not converge on a single terminal phenotype, but instead diversify into multiple late-stage states with distinct functional properties (44). Trajectory reconstruction identified intermediate populations linking quiescent HSCs to terminal fibrogenic states, while late-stage HSCs exhibited enrichment of pathways related to ECM organization, cellular migration, inflammatory signaling, and metabolic stress responses. These findings indicate that functional specialization emerges progressively along the activation trajectory, suggesting that late-stage fibrosis is maintained by heterogeneous HSC populations rather than a uniform myofibroblast pool (39).

Evidence for non-linear and branched HSC trajectories was further strengthened by Bai et al., who demonstrated that quiescent HSCs can diverge into multiple activation paths under fibrotic stress (43). One trajectory was dominated by matrix remodeling and fibrogenic programs, whereas an alternative trajectory was characterized by enhanced immune-regulatory and chemotactic activity. In addition, a proliferative trajectory was identified, suggesting that proliferation may contribute to amplification of fibrogenic cell populations. Collectively, these observations support a model in which HSC activation is directionally flexible, with distinct trajectories potentially reflecting differences in injury context, microenvironmental cues, or disease stage (86).

Crucially, trajectory patterns identified in animal models are conserved in human liver fibrosis, underscoring their translational relevance. In scRNA-seq analyses of fibrotic human liver tissue, Ai et al. demonstrated that HSCs undergo transdifferentiation toward myofibroblast-like states during disease progression (45). This transition recapitulates terminal fibrogenic states inferred from mouse models, indicating that pseudotime-defined HSC trajectories capture biologically and clinically meaningful processes rather than model-specific phenomena. The cross-species consistency of these activation routes provides strong support for the use of experimental models to dissect regulatory mechanisms governing human fibrogenesis (49).

From a clinical and translational perspective, trajectory-based insights into HSC activation have several important implications. First, the identification of intermediate activation states suggests the existence of therapeutic windows during which HSCs may remain amenable to reprogramming or deactivation (112). Second, the presence of branched trajectories implies that fibrosis progression may be driven by distinct fibrogenic programs in different patient subsets, offering a potential explanation for variability in disease course and treatment response (107). Finally, trajectory-informed molecular programs may enable the development of pathway-based biomarkers for patient stratification, disease staging, and monitoring of antifibrotic therapies (113). Together, these findings position HSC trajectory analysis as a critical conceptual and methodological framework for advancing precision medicine approaches in liver fibrosis (114).

### 4.2 Macrophage trajectories and fibrogenic polarization

Macrophages constitute a highly plastic and heterogeneous immune compartment in the fibrotic liver, and trajectory-based single-cell analyses have been instrumental in redefining their roles beyond static M1/M2 classification schemes (115). Pseudotime reconstruction has revealed that macrophage states in liver injury are shaped by dynamic differentiation trajectories and polarization shifts, driven by continuous exposure to inflammatory cues, tissue damage signals, and fibrotic microenvironments. These insights underscore macrophages as both initiators and sustainers of fibrogenesis through temporally coordinated functional transitions (67). In murine toxic injury models, trajectory analyses demonstrate that infiltrating monocytes are rapidly recruited to the injured liver and initially adopt pro-inflammatory programs that amplify tissue damage. Along pseudotime, these cells progressively mature into monocyte-derived macrophages with reduced inflammatory output and enhanced involvement in tissue remodeling (116). Using a CCl_4_-induced fibrosis model, Cheng et al. captured this maturation trajectory, highlighting a shift from early inflammatory signaling toward pathways associated with immune regulation and fibrogenesis (42).

Macrophage trajectories also encompass polarization-driven state transitions. In TCS-induced mouse liver injury, Bai et al. showed that resident Kupffer cells and liver capsular macrophages undergo a functional reprogramming toward profibrotic polarization states, accompanied by proliferative expansion (43). This process reflects dynamic polarization rather than classical lineage differentiation, emphasizing the reversible and context-dependent nature of macrophage states in chronic liver injury.

A major advance was the identification of SAMs as fibrosis-associated cell states. In combined toxic and cholestatic mouse models, Yang et al. inferred that bone marrow–derived macrophages transition into SAMs along a defined pseudotime axis during fibrosis progression (62). This trajectory was characterized by enrichment of extracellular matrix organization, angiogenesis, wound healing, and fibroblast activation pathways, and was largely absent in healthy livers, underscoring its fibrosis-specific nature.

Human single-cell studies corroborate these findings. In HBV-associated fibrosis, Bai et al. reconstructed myeloid trajectories showing a directional shift from immature monocytes toward mature macrophage and dendritic cell states, accompanied by enhanced antigen presentation and inflammatory pathway activity (63). Seminal work by Ramachandran et al. further demonstrated that scar-associated macrophages in human cirrhosis derive from circulating monocytes and localize to fibrotic niches, exhibiting conserved fibrogenic programs across species (23).

Macrophage trajectory analyses have important translational implications. First, they suggest that therapeutic timing is critical: early intervention targeting monocyte recruitment or inflammatory macrophage states may limit injury amplification, whereas later-stage strategies aimed at disrupting SAM differentiation or profibrotic polarization may attenuate fibrosis maintenance (117). Second, trajectory-defined macrophage states provide a rationale for pathway-based biomarkers reflecting immune remodeling rather than absolute cell abundance. Finally, the identification of conserved fibrogenic macrophage trajectories across etiologies and species highlights macrophages as attractive targets for broad-spectrum antifibrotic therapies (104). Together, these studies position macrophage trajectories as a central component of systems-level models of liver fibrosis progression (118, 119).

### 4.3 Liver sinusoidal endothelial cells trajectories and vascular remodeling

LSECs in the liver exhibit remarkable plasticity during fibrosis, with single-cell trajectory analyses providing critical insights into the mechanisms of sinusoidal remodeling and capillarization (120). Pseudotime reconstruction has revealed that LSECs progressively lose their specialized fenestrated phenotype and acquire capillary-like features, a hallmark of endothelial dysfunction in chronic liver disease (71). This dynamic reprogramming links vascular changes directly to fibrogenic progression and the modulation of hepatic microenvironmental cues (121).

In murine models of drug-induced fibrosis, Bai et al. demonstrated that pericentral and select midzonal LSECs transition toward central vein–associated endothelial cell states (LVEC_central), accompanied by downregulation of classical sinusoidal markers and upregulation of ECM-related programs (43). Transcription factor analysis identified Hlf (Hepatocyte Leukemia Factor) as a key regulator driving this phenotypic shift, highlighting potential molecular targets for intervention. Human data, though limited, suggest conservation of these capillarization programs, with dysfunctional LSECs showing ECM upregulation and enhanced interactions with activated HSCs and inflammatory macrophages.

Clinically, understanding EC trajectory dynamics has direct implications. Early intervention to disrupt capillarization may preserve sinusoidal function, maintain nutrient and oxygen exchange, and attenuate HSC activation (122). Targeting endothelial-derived signals at later stages could limit microvascular remodeling and indirectly reduce fibrosis progression (123). Overall, trajectory-informed analyses position vascular remodeling as both a marker and mediator of liver fibrosis, providing avenues for mechanism-based therapies and predictive biomarkers (124).

### 4.4 B lymphocyte trajectories and non-canonical fibrogenic functions

B lymphocytes, traditionally recognized for their adaptive immune roles, have recently been shown to exhibit dynamic trajectory patterns in liver fibrosis, highlighting previously underappreciated contributions to the fibrotic microenvironment (125). Single-cell pseudotime analyses reveal that B lymphocytes undergo progressive differentiation from naïve states toward both plasma and memory fates, reflecting lineage diversification and functional specialization during chronic liver injury (126). Importantly, these trajectories suggest that B lymphocytes not only participate in classical antibody-mediated immunity but may also influence fibrogenesis through interactions with stromal and myeloid compartments (127).

In murine TCS-200–induced fibrosis, Bai et al. demonstrated that B_naive cells differentiate along two primary trajectories toward B_plasma or B_memory subsets, with the latter accompanied by a minor plasma population (43). Despite an overall reduction in total B-cell proportion, these trajectories indicate active lineage commitment and potential recruitment to fibrotic niches. Memory and plasma branches may modulate macrophage activation, HSC behavior, and cytokine networks, revealing non-canonical profibrotic functions of B lymphocytes.

Although human single-cell B-cell trajectories are less explored, evidence suggests conserved principles: memory and plasma subsets accumulate in fibrotic regions and secrete pro-inflammatory and profibrotic cytokines that can indirectly activate HSCs and ECM deposition (128). Clinically, naïve B-cell depletion and effector/memory expansion could serve as biomarkers of immune exhaustion or fibrosis progression (129). Interventions targeting B-cell differentiation or functional output may modulate profibrotic immune circuits without broadly suppressing adaptive immunity, establishing B lymphocytes as complementary contributors to fibrogenesis (130).

### 4.5 Integrated multicellular trajectories and translational implications

The pseudotime–based mapping of hepatic cell populations—including HSCs, macrophages, LSECs, and B lymphocytes—provides a comprehensive framework for understanding the dynamic orchestration of liver fibrosis (23). Across both animal models and human studies, these analyses reveal that fibrosis is not driven by isolated cell types but emerges from temporally and spatially coordinated transitions across multiple compartments (131). HSCs progressively activate from quiescent to fibrogenic effector states, macrophages shift from inflammatory monocytes to scar-associated subsets, LSECs undergo capillarization, and B lymphocytes differentiate into memory and plasma lineages, collectively remodeling the hepatic microenvironment (31).

Integrating these trajectories highlights convergent nodes of fibrogenic activity. For example, HSC activation is tightly coupled to macrophage maturation and polarization, with cytokines, growth factors, and ECM components forming feedback loops that reinforce fibrogenesis (132). Endothelial remodeling further modulates both immune cell recruitment and HSC activation through altered vascular permeability and paracrine signaling (77). Meanwhile, B lymphocytes, through effector and memory differentiation, contribute to adaptive immune remodeling, shaping macrophage and HSC behavior via cytokine secretion (41). This multi-compartment crosstalk suggests that fibrosis represents a systems-level disease, where therapeutic targeting of single pathways in isolation may be insufficient (133).

From a translational perspective, the integrated multicellular trajectory framework offers several key insights. First, it identifies stage-specific intervention windows: early inhibition of monocyte recruitment or naïve B-cell activation may limit inflammatory amplification, while modulation of HSC terminal differentiation or endothelial capillarization could attenuate ECM deposition and vascular dysfunction (77). Second, trajectory-defined cell states enable pathway-based biomarker development, providing readouts of fibrogenic activity that are more precise than bulk cell counts or single markers (134). Third, conserved trajectories between animal models and human patients validate preclinical targets and facilitate rational design of broad-spectrum antifibrotic strategies that simultaneously address multiple pathogenic cell populations (135).

The convergence of HSC, macrophage, endothelial, and B-cell trajectories underscores the systems-level logic of liver fibrosis (136). By mapping dynamic cellular states and intercellular communication, single-cell trajectory analyses not only advance mechanistic understanding but also inform translational strategies for early detection, patient stratification, and combinatorial therapeutic intervention, establishing a foundation for precision antifibrotic medicine (134).

## 5 Cell–cell communication networks orchestrating liver fibrogenesis

Liver fibrosis arises from coordinated interactions among stromal, immune, endothelial, and parenchymal cells rather than from isolated cell-intrinsic programs (117). With the application of single-cell transcriptomics and ligand–receptor inference frameworks, it has become possible to quantitatively reconstruct intercellular communication networks and reveal how these interactions are dynamically rewired during fibrogenesis (137). Analyses of multiple experimental models consistently show that fibrosis is associated with a marked increase in both the number and strength of cell–cell interactions, with HSCs and macrophages exhibiting the highest connectivity and signaling activity compared with other cell populations (117). In CCl_4_- and diet- or toxin-induced fibrosis models, these two cell types display a disproportionate expansion of outgoing and incoming signaling, particularly involving pathways related to extracellular matrix remodeling, inflammatory cytokine signaling, and growth factor–mediated activation (132). Key ligand–receptor axes—including TGF-β, PDGF, CCL–CCR, and integrin-mediated ECM signaling—emerge as central regulators linking tissue injury sensing to fibrogenic activation and progression (137, 138).

Importantly, human single-cell studies of HBV-related cirrhosis and NASH further validate these findings, demonstrating that HSCs and macrophages remain dominant communication hubs in diseased livers, with conserved enrichment of profibrotic and immunoregulatory signaling pathways (23). Moreover, stage-dependent remodeling of communication networks is observed, with early disease characterized by immune-driven signaling and later stages dominated by matrix deposition and vascular adaptation programs (137). Cross-species comparisons indicate that these interaction patterns are largely conserved despite differences in etiology, supporting the robustness of communication-defined fibrogenic programs (87).

Collectively, these findings highlight that liver fibrosis is driven by dynamic and hierarchical intercellular communication networks rather than static cellular composition (48). From a translational perspective, targeting key communication nodes—rather than individual cell types—may enable simultaneous modulation of inflammation, extracellular matrix deposition, and vascular remodeling, thereby improving therapeutic efficacy. In addition, communication-derived molecular signatures provide a framework for biomarker development and patient stratification based on pathway activity.

**Figure 3** summarizes selected communication pathways among HSCs, macrophages, and LSECs. Different line styles distinguish predicted interactions, functional evidence, and predictions accompanied by functional support. Support for a mediator or pathway does not establish every source-specific ligand–receptor pair *in vivo*. The cell–cell communication features summarized in **Supplementary Table S3** (139).

**Figure 3:**
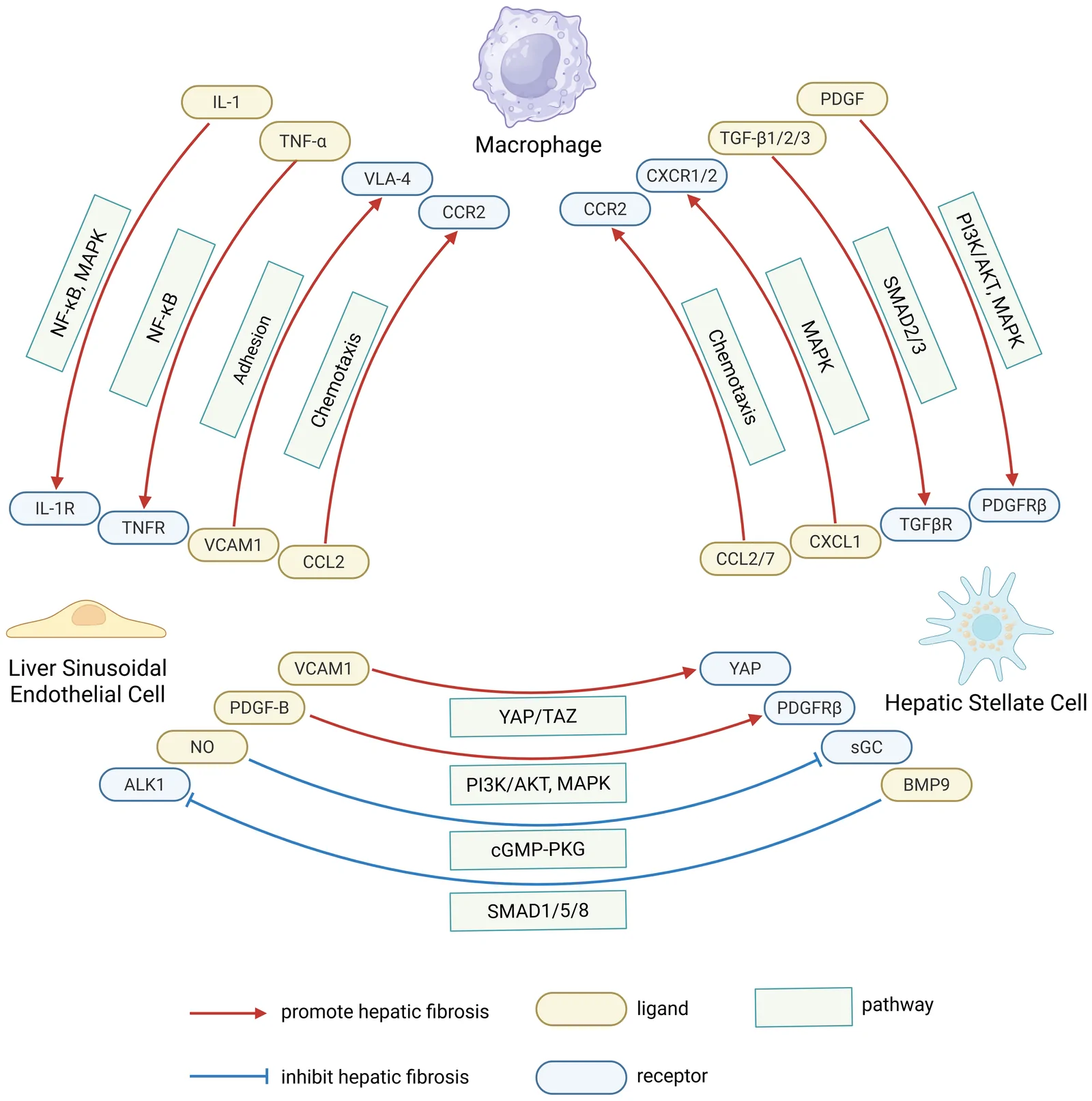
Conceptual schematic of intercellular communication among hepatic stellate cells (HSCs), macrophages, and liver sinusoidal endothelial cells (LSECs) during liver fibrosis. Yellow boxes indicate ligands, blue boxes indicate receptors, and teal boxes represent intracellular signaling pathways. The schematic summarizes reported and predicted ligand–receptor interactions from single-cell studies and functional evidence. Red and blue lines indicate profibrotic and protective associations, respectively, rather than direct causal effects. Line color reflects the biological direction of the interaction rather than the type or level of experimental evidence, and functional support for a mediator or pathway does not necessarily indicate that every specific ligand–receptor pair has been validated in vivo. This figure was created with BioRender.

### 5.1 Hepatic stellate cell–centered communication networks in liver fibrosis

HSCs occupy a central position in the fibrotic microenvironment, not only as the principal source of extracellular matrix but also as highly active signaling hubs that coordinate multicellular responses to chronic liver injury (140). Cell–cell communication analyses based on single-cell transcriptomic data have revealed that HSCs engage in extensive and dynamic interactions with immune, endothelial, and stromal compartments, enabling amplification and stabilization of profibrotic programs across the tissue (44).

In experimental fibrosis models, multiple studies have consistently identified HSCs as the most interconnected cellular population within the hepatic niche. Using a CCl_4_-induced mouse model, Cheng et al. demonstrated that HSCs constitute the most trophic and densely connected cell type in the liver, suggesting their dominant role in orchestrating intercellular signaling during fibrosis (42). Complementarily, in a TCS-200–induced fibrosis model, Bai et al. showed that activated HSC subsets—particularly aHSC1—exhibited the highest number of interactions with other cell populations, positioning them as central communicators that integrate inflammatory, vascular, and matrix-related cues (43).

Further refinement of HSC heterogeneity has revealed functional specialization within HSC-centered communication networks. In CCl_4_-treated mice, Zhang et al. identified distinct HSC subpopulations with divergent signaling roles: one subset acting as a key regulator of inflammation and extracellular matrix deposition during injury-induced remodeling, and another associated with tissue repair, vasoactive responses, and angiogenesis (44). These findings suggest that HSC-mediated communication is not uniform but temporally and functionally stratified, allowing HSCs to coordinate both fibrogenic and compensatory repair processes (35). Importantly, human data support the translational relevance of these observations. In fibrotic human liver samples, Ai et al. demonstrated that ligand–receptor interactions between HSCs and myofibroblasts form a coordinated communication module that facilitates HSC-to-myofibroblast transition, reinforcing fibrogenic signaling circuits in advanced disease (45).

Recognition of HSCs as communication hubs reframes their role from passive ECM producers to active signal integrators and amplifiers. Disrupting HSC-centered communication networks—rather than targeting HSC activation alone—may allow simultaneous modulation of inflammation, angiogenesis, and matrix remodeling (141). Such strategies hold promise for attenuating fibrosis progression while minimizing off-target effects, and HSC-derived communication signatures may serve as biomarkers for disease activity and therapeutic response (139).

### 5.2 Macrophage-driven communication circuits in fibrotic liver remodeling

Macrophages are highly plastic immune cells that adapt their communication profiles in response to chronic liver injury, enabling them to act as key intermediaries between inflammation, fibrogenesis, and tissue remodeling (117, 142). Cell–cell communication analyses have revealed that fibrotic macrophages actively shape the hepatic microenvironment by delivering profibrotic signals to stellate, endothelial, and mesenchymal cells, thereby reinforcing pathogenic cellular states (56).

In mouse models of CCl_4_-induced fibrosis, Cheng et al. demonstrated that fibrotic macrophages secrete a broad repertoire of growth factors, chemokines, and adhesion molecules that promote HSC proliferation, activation, and extracellular matrix remodeling (42). Beyond paracrine support, THBS1-dependent HSC activation has functional support, although the proposed integrin receptor assignments remain incompletely resolved (42, 143). These findings position macrophages as active drivers—rather than secondary responders—of stellate cell–dependent fibrosis.

The importance of macrophage-centered communication is further underscored by studies focusing on SAMs. Using both CCl_4_- and bile duct ligation–based fibrosis models, Yang et al. identified Plg-RKT–dependent signaling as a critical axis promoting the transformation of bone marrow–derived macrophages into SAMs, highlighting a communication mechanism that links macrophage lineage remodeling to fibrotic niche formation (62). Human studies corroborate these observations. In HBV-related cirrhosis, Bai et al. reported enhanced communication between myeloid cells and multiple immune populations, indicating that macrophages function as communication amplifiers within the immune network of fibrotic livers (63).

At the tissue level, large-scale ligand–receptor analyses in cirrhotic human liver have revealed extensive intercellular communication among expanded pathogenic cell populations (144). Ramachandran et al. demonstrated that SAMs engage in dense signaling exchanges with scar-associated endothelial and mesenchymal cells, promoting mesenchymal proliferation and fibrotic expansion through coordinated receptor-mediated pathways (23). These findings suggest that macrophage-driven communication networks underpin the stability and persistence of the fibrotic niche in advanced disease.

Macrophage-centered communication circuits represent attractive therapeutic targets. Intervening at key macrophage signaling interfaces may simultaneously dampen inflammation, limit HSC activation, and restrain mesenchymal expansion (145). Moreover, macrophage-derived communication signatures could aid in identifying patients with active fibrogenic remodeling and guide the development of immune-modulatory antifibrotic therapies (146).

### 5.3 Hepatic stellate cell–macrophage crosstalk in liver fibrosis: inflammatory–fibrogenic coupling

HSCs and macrophages constitute a core multicellular axis that drives the initiation and progression of liver fibrosis (132, 147). Beyond their individual roles in extracellular matrix production and immune regulation, these two cell types engage in tightly coordinated bidirectional communication that links tissue injury sensing to fibrogenic activation (148). Single-cell transcriptomic studies consistently demonstrate that HSCs and macrophages represent the most highly connected and interactive populations within the fibrotic niche, forming a central signaling circuit that integrates inflammatory cues, growth factor signaling, and extracellular matrix remodeling (42). This reciprocal crosstalk not only amplifies local inflammatory responses but also stabilizes pathogenic cell states, thereby sustaining fibrosis progression across disease stages (119, 149).

From the perspective of HSC-to-macrophage signaling, multiple studies have identified activated HSC subsets as key sources of chemokine-driven immune recruitment and modulation. Analyses from Yang and Zhang in murine CCl_4_ and bile duct ligation models revealed that inflammatory HSC subpopulations, particularly HSC3-like clusters, exhibit strong expression of chemokines such as CCL2 and CCL7, which interact with candidate chemokine receptors, including CCR2, on macrophages to promote monocyte recruitment and macrophage accumulation (31, 44). In parallel, these HSC subsets also produce CXCL1, engaging CXCR2 signaling to regulate neutrophil-associated inflammatory responses, further shaping the immune microenvironment. Extending these findings to human disease, Bai demonstrated the presence of CCL5^+^ macrophage populations associated with chemokine-rich HSC niches, supporting a conserved role for HSC-derived chemokine signaling in orchestrating immune cell infiltration (63). Collectively, these results highlight chemokine signaling pathways as dominant mediators of HSC-driven immune activation, positioning HSCs as upstream regulators of macrophage recruitment, polarization, and inflammatory amplification (150).

Conversely, macrophage-to-HSC communication represents a major effector arm that directly promotes fibrogenic activation. Multiple independent studies—including those by Yang, Cheng, Bai, and Ramachandran—consistently show that macrophages establish high-intensity signaling interactions with activated HSC subsets, particularly aHSC1-like populations, Kupffer cell–derived macrophages, and monocyte-derived macrophage clusters. Among these, TGF-β signaling emerges as the most prominent profibrotic pathway, with macrophage-derived TGFβ1/2/3 engaging TGFβ receptors on HSCs to drive myofibroblastic differentiation and extracellular matrix production (31, 42, 43). In addition, PDGF–PDGFR signaling has been identified as a critical proliferative axis, particularly in scar-associated macrophage niches described in human cirrhotic livers, where macrophages promote HSC expansion and survival; the human study supports a PDGFRA-related axis (23, 42). Proinflammatory signaling pathways, including Tumor necrosis factor–tumor necrosis factor receptor (TNF–TNFR) and Interleukin-1β–interleukin-1 receptor (IL1B–IL1R) axes, further contribute to sustaining HSC activation and amplifying fibrogenic responses (42, 43). Chemokine signaling also operates in the reverse direction, with macrophage-derived CCL2 reinforcing HSC–immune cell feedback loops (23, 31, 43). Notably, these interactions are not uniformly distributed but are concentrated within specific cellular interfaces, including macrophage–aHSC clusters and scar-associated macrophage–mesenchymal niches, indicating spatially restricted yet functionally dominant communication hubs.

The integrated HSC–macrophage communication network provides a compelling framework for therapeutic intervention (151). Targeting key signaling axes within this bidirectional circuit—such as CCR2-mediated monocyte recruitment, TGF-β–driven fibrogenic activation, or PDGF-dependent HSC proliferation—may enable simultaneous modulation of inflammation and matrix deposition. Importantly, these pathways have already been explored in preclinical and early clinical studies, although clinical benefit has not been consistently demonstrated (see Section 6.4) (152). Furthermore, communication-derived signatures, including chemokine expression profiles and ligand–receptor interaction strength, may serve as biomarkers for disease stage, inflammatory activity, and treatment response (153). By shifting the focus from single-cell targeting to network-level intervention, HSC–macrophage crosstalk represents a promising entry point for the development of more effective and precise antifibrotic therapies (117).

### 5.4 Endothelial–stellate cell crosstalk in liver fibrosis: angiocrine regulation and fibrogenic reprogramming

Hepatic stellate cells and liver sinusoidal endothelial cells form a highly specialized and interdependent unit within the hepatic sinusoidal microenvironment, in which endothelial-derived angiocrine signals critically regulate stellate cell activation states and fibrogenic potential. Under physiological conditions, differentiated LSECs function as key anti-fibrotic gatekeepers that actively maintain HSC quiescence and even promote reversion of activated HSCs to a quiescent phenotype (78). This regulatory effect is largely mediated by endothelial nitric oxide (NO), as reduced NO production in cirrhotic livers directly promotes HSC activation and contractility (154). In parallel, intact endothelial differentiation and angiocrine signaling are required to preserve sinusoidal homeostasis and restrain fibrogenesis, highlighting the central role of LSECs in maintaining the non-fibrotic hepatic niche (155, 156).

A critical pathogenic switch occurs during chronic liver injury, when LSECs undergo capillarization, characterized by loss of fenestrae, deposition of basement membrane, and altered expression of adhesion molecules and matrix proteins (157, 158). This endothelial dedifferentiation disrupts angiocrine homeostasis and abolishes the ability of LSECs to control HSC quiescence, thereby promoting stellate cell activation and fibrosis progression (159). Mechanistically, multiple signaling pathways converge to drive LSEC capillarization, including Hedgehog signaling and DLL4-mediated Notch activation, both of which promote endothelial dysfunction and fibrogenesis. In addition, immune-mediated mechanisms, such as endothelial autoantibody-induced capillarization and altered immunogenicity of LSECs, further exacerbate endothelial dysfunction and contribute to fibrotic remodeling (158, 160).

Following capillarization, LSECs acquire a pro-fibrotic phenotype and actively promote HSC activation through diverse angiocrine and signaling mechanisms. Endothelial-derived A-FABP drives HSC activation via JNK/c-Jun-dependent TGF-β signaling (161), while LSEC-derived VCAM1 promotes HSC activation in experimental models and is associated with YAP1 activation in cultured human HSCs. The receptor mechanism linking VCAM1 to this intracellular response remains incompletely resolved (162). Endothelial transcriptional programs also play a pivotal role, as GATA4-dependent endothelial differentiation restrains fibrogenesis, whereas its disruption induces PDGF-B expression and promotes HSC activation (156, 163). Moreover, metabolic and mechanotransduction pathways further amplify this crosstalk, as glycolysis-dependent angiocrine signaling in LSECs enhances fibrogenic responses under conditions of increased matrix stiffness (164).

Notably, this communication is bidirectional and dynamically regulated. HSC-derived BMP9 supports LSEC fenestration and endothelial differentiation in the experimental settings studied, including 129/Ola mice and cultured LSECs (165). Its net effect should not be assumed to be uniformly protective across strains or injury contexts. In addition, extracellular vesicle (EV)-mediated signaling adds another layer of complexity, as LSECs-derived exosomes can promote HSC migration and activation through sphingosine-1-phosphate–dependent pathways (166), whereas more recent evidence indicates that LSEC-derived EVs may also exert inhibitory effects on HSC activation, suggesting context-dependent regulatory functions (167). Collectively, HSC–LSEC communication undergoes a profound shift from a homeostatic, angiocrine-regulated anti-fibrotic network to a pathogenic, self-amplifying circuit characterized by endothelial capillarization, pro-fibrotic signaling, and metabolic reprogramming, ultimately driving liver fibrosis progression.

### 5.5 Macrophage–endothelial crosstalk in liver fibrosis: immunogenic reprogramming and inflammatory amplification

Kupffer cells (KCs) and LSECs form a tightly coupled immunovascular interface that orchestrates hepatic immune surveillance, inflammatory responses, and fibrogenesis. Under physiological conditions, LSECs maintain immune tolerance by regulating leukocyte trafficking and limiting excessive immune activation within the sinusoidal environment, thereby contributing to hepatic immunological homeostasis (124, 168). This tolerogenic phenotype is supported by controlled interactions with macrophages, ensuring balanced immune responses to circulating antigens and microbial products.

During chronic liver injury, however, LSECs undergo profound phenotypic reprogramming and acquire enhanced immunogenicity, thereby shifting from a tolerogenic to a pro-inflammatory state (160). Capillarized and activated LSECs exhibit increased expression of adhesion molecules and cytokines, facilitating leukocyte adhesion, transmigration, and local immune activation (157). In parallel, macrophage-derived inflammatory mediators further exacerbate endothelial activation, creating a feed-forward loop that amplifies hepatic inflammation and promotes fibrosis progression.

This bidirectional crosstalk is further shaped by endothelial-derived signals that regulate macrophage recruitment and activation. LSECs modulate chemokine gradients and sinusoidal permeability to control monocyte infiltration, while angiocrine signaling influences macrophage polarization and function (169). In addition, endothelial dysfunction driven by impaired autophagy contributes to enhanced inflammatory signaling and fibrogenesis, highlighting the importance of intracellular regulatory pathways in macrophage–endothelial interactions (170).

Emerging evidence also suggests that extracellular vesicle-mediated communication plays a role in macrophage–endothelial crosstalk. LSECs-derived EVs can suppress activation of both HSCs and Kupffer cells, indicating a potential anti-inflammatory and anti-fibrotic mechanism within the sinusoidal niche (167). Moreover, immune-mediated signaling pathways, including Th1/Th2-driven modulation of endothelial phenotype via Rho–ROCK–myosin signaling, further contribute to capillarization and inflammatory amplification (171).

Overall, macrophage–endothelial crosstalk represents a central immunovascular hub that integrates endothelial dysfunction, immune activation, and fibrogenic signaling. While this axis maintains immune tolerance under physiological conditions, its dysregulation during chronic liver injury leads to sustained inflammation, endothelial activation, and amplification of pro-fibrotic pathways, ultimately contributing to the progression of liver fibrosis.

## 6 Challenges from single-cell discovery to clinical translation

Despite the substantial insights provided by single-cell transcriptomic studies into the cellular heterogeneity and intercellular communication underlying liver fibrosis, several limitations should be considered when interpreting the current body of evidence (172).

### 6.1 Platform choice and tissue recovery

Technical constraints associated with single-cell RNA sequencing may introduce biases in the identification and quantification of specific cell populations. The enzymatic dissociation required for single-cell analysis can lead to the preferential loss of fragile or tightly embedded cells within fibrotic tissue, particularly extracellular matrix–producing stromal populations (173). In addition, transcriptional dropout and variability in sequencing depth may affect the accurate detection of low-abundance transcripts, potentially influencing the classification of rare or transitional cell states involved in fibrogenesis (26).

Platform choice further affects these results. Droplet-based 10x Chromium supports high throughput, whereas plate-based Smart-seq2 generally detects more genes per cell and provides full-length coverage at lower throughput (174, 175). Cell recovery and transcript detection are distinct: low recovery can obscure rare populations, while limited depth can miss low-abundance ligands or receptors. Neither recovered cell fractions nor absent transcripts directly establish tissue abundance or biological absence.

Single-nucleus RNA sequencing (snRNA-seq) permits analysis of frozen tissue and reduces dependence on viable-cell dissociation, making it useful for dense fibrotic samples and archived cohorts. However, nuclear RNA differs from whole-cell RNA, with reduced representation of some cytoplasmic transcripts and a greater contribution from unspliced RNA. A matched human-liver study demonstrated complementary recovery by scRNA-seq and snRNA-seq, but used healthy tissue; equivalent performance cannot be assumed in cirrhosis (176). Spatial or histological validation remains necessary.

### 6.2 Trajectory and communication inference

Many conclusions regarding cellular differentiation trajectories during liver fibrosis are derived from computational pseudotime inference rather than direct lineage tracing. Although trajectory analysis has been widely used to reconstruct activation pathways of HSCs or the differentiation of monocyte-derived macrophages into pro-fibrotic phenotypes, these models rely on transcriptional similarity and therefore represent inferred developmental relationships (177). Experimental validation through lineage-tracing systems, functional perturbation studies, or longitudinal sampling will be necessary to confirm these proposed cellular transitions (178).

Current analyses of intercellular communication in liver fibrosis are largely based on computational inference of ligand–receptor interactions from single-cell transcriptomic datasets (179). While these approaches provide valuable hypotheses regarding signaling networks between HSCs, macrophages, endothelial cells, and other immune populations, transcriptional co-expression does not necessarily indicate active signaling *in vivo* (180). Additional experimental approaches, including spatial validation and functional assays, are required to determine whether predicted communication pathways are biologically relevant drivers of fibrogenesis (181).

### 6.3 Limits of cross-species integration

Importantly, given the qualitative, narrative nature of this review, several limitations related to cross-species integration should be acknowledged. The heterogeneity of bioinformatic pipelines across individual studies—including differences in data preprocessing, clustering strategies, and cell-type annotation—may introduce variability and noise into cross-dataset and cross-species comparisons (182). Moreover, reliance on pathway-level annotations may obscure underlying biological differences, as identically named pathways in human and mouse datasets may be driven by distinct orthologous gene sets, potentially affecting the interpretation and translational relevance of conserved signaling programs (183).

Biological differences also matter: toxin-induced injury, biliary obstruction, and metabolic disease differ in injury distribution, duration, and immune context. Shared markers therefore do not guarantee equivalent cell functions or treatment responses. One-to-one ortholog mapping can discard species-specific genes, while aggressive batch correction may remove meaningful biological differences (184). Comparisons should retain donor-level replication, assess both shared and divergent pathway-driving genes, and test candidate mechanisms in relevant human tissue. Reused datasets, such as GSE136103 in Ai et al. and Ramachandran et al., are not independent cohorts (23, 45). This review compares published results qualitatively rather than constructing a joint human–mouse expression atlas.

Most available single-cell datasets are generated from limited numbers of patient samples or experimental models, which may not fully capture the heterogeneity of liver fibrosis across different etiologies, disease stages, or clinical contexts (180). Importantly, the human studies emphasized here do not cover all major etiologies equally; broader representation of alcohol-associated liver disease (ALD), HCV infection, and autoimmune liver diseases remains necessary. Expanding cohort sizes and integrating datasets across multiple studies will therefore be critical for improving the robustness and reproducibility of identified cellular programs (185). Furthermore, translating high-resolution molecular insights into clinically actionable biomarkers or therapeutic targets remains an important challenge that requires closer integration between experimental research and clinical investigation (181).

### 6.4 Clinical setbacks and therapeutic translation

Mechanistic plausibility does not ensure clinical efficacy. In the phase III AURORA trial, the dual CCR2/CCR5 antagonist cenicriviroc did not improve fibrosis without worsening steatohepatitis compared with placebo (22.3% versus 25.5% at month 12; P = 0.21) (186). This result challenges direct translation of chemokine-pathway hypotheses but is not a selective test of CCL2 alone. TGF-β inhibition raises additional selectivity and safety concerns: fresolimumab-associated keratoacanthomas and squamous-cell carcinomas were reported in oncology, not in a liver-fibrosis efficacy trial (187). Antifibrotic galunisertib findings in human liver slices remain ex vivo evidence (188).

Single-cell profiling may help identify responsive cell states and compensatory pathways through paired pretreatment and on-treatment sampling. Spatial protein measurements and perturbation experiments are then needed to establish target engagement and causal effects. These applications could improve patient and target selection, but have not yet been shown to explain or overcome these clinical setbacks.

### 6.5 Biomarker feasibility and established non-invasive tests

Cell-state markers require validation as clinical assays. CD9 and ACTA2 are expressed in multiple cell populations, so cluster-level enrichment cannot establish patient-level diagnostic specificity. Circulating TREM2 has preliminary clinical evidence: in a validation cohort of 170 patients with liver stiffness ≥8.0 kPa, the endpoint was biopsy-assessed NAFLD activity score (NAS) ≥4, termed NASH in the original study. Plasma TREM2 thresholds of ≤38 ng/mL (rule-out) and ≥65 ng/mL (rule-in) had sensitivity/specificity of 90%/52% and 54%/89%, respectively (189). These estimates are cohort- and endpoint-specific; the same study reported an AUROC of only 0.61 for advanced fibrosis (F3–F4 versus F0–F2) (189). Experimental evidence that TREM2 can limit injury also cautions against treating marker enrichment as a rationale for inhibition (190).

FIB-4 uses routine age, aminotransferase, and platelet measurements, whereas ELF combines markers of extracellular matrix turnover; both have established roles in fibrosis risk assessment (191). New signatures must be compared with these tests in independent cohorts using the same endpoint, reference standard, and prespecified thresholds. Sensitivity, specificity, predictive values, calibration, assay reproducibility, cost, and added clinical benefit should be assessed. Incremental value over FIB-4 or ELF for fibrosis detection or risk stratification remains to be demonstrated.

Taken together, while single-cell transcriptomic technologies have significantly advanced our understanding of the multicellular mechanisms driving liver fibrosis, continued methodological refinement, larger patient cohorts, and rigorous experimental validation will be essential to strengthen the biological and translational significance of these findings (105).

## 7 Future perspectives

The rapid development of single-cell transcriptomics has fundamentally transformed our understanding of the cellular heterogeneity and dynamic interactions that drive liver fibrosis (192). Importantly, current insights into liver fibrosis are derived from both human cohort studies and experimental animal models, which represent different levels of evidence: human studies provide direct clinical relevance but are often limited by sample accessibility and inter-individual heterogeneity, whereas animal models (e.g., CCl_4_- and BDL-induced fibrosis) enable controlled mechanistic investigation and functional validation but may not fully recapitulate the complexity of human disease. Therefore, future efforts should focus on integrating mechanistic discoveries from animal models with validation in human cohorts to establish a more robust and translational framework for liver fibrosis research. Nevertheless, future progress in the field will depend on the integration of complementary technologies that can bridge molecular insights with spatial context and clinical relevance (193).

One of the most promising future directions in liver fibrosis research is the integration of single-cell RNA sequencing with spatial transcriptomic technologies (194). While single-cell approaches have greatly improved the resolution at which cellular heterogeneity and differentiation trajectories can be characterized, they inherently lack information about the spatial organization of cells within the tissue microenvironment (195). Spatial transcriptomics addresses this limitation by preserving tissue architecture and enabling transcriptomic profiling directly within histological sections, thereby providing critical spatial context for interpreting cell states identified by single-cell analyses (196, 197). Emerging studies have begun to illustrate the value of this integrative framework in fibrotic liver disease. Single-cell transcriptomic analyses have revealed distinct fibrogenic cell populations, including activated HSCs and scar-associated macrophages, which are thought to cooperate within specialized fibrotic niches (23). Complementary spatial transcriptomic studies further indicate that fibrotic regions exhibit distinct transcriptional landscapes enriched for mesenchymal and immune cell populations, suggesting that fibrogenesis is orchestrated within spatially organized multicellular microenvironments (198). In addition, single-cell analyses have demonstrated that HSCs display zonated transcriptional programs across the liver lobule, highlighting the importance of spatial context in determining fibrogenic potential (104). The continued integration of single-cell and spatial transcriptomic approaches is therefore expected to provide a more comprehensive understanding of how fibrogenic cell populations interact within the liver microenvironment (21). By combining cellular identity, transcriptional state, and spatial localization, future studies may more accurately define pathogenic cell–cell communication networks and identify spatially restricted signaling hubs that drive fibrosis progression. Such insights may ultimately facilitate the development of more precise therapeutic strategies targeting specific cellular niches within fibrotic liver tissue (199).

Beyond transcriptomics, emerging multi-omics technologies offer additional opportunities to deepen our understanding of fibrogenesis (200). Integrative analyses combining single-cell transcriptomics with single-cell epigenomics, proteomics, and metabolomics could reveal regulatory mechanisms that control cell-state transitions during fibrosis. For example, single-cell chromatin accessibility profiling may help identify transcription factors and regulatory elements that drive HSC activation, while spatial proteomic platforms could provide complementary insights into protein-level signaling within fibrotic microenvironments (201). Furthermore, advances in computational modeling and machine learning are expected to play an increasingly important role in integrating large-scale multi-modal datasets and reconstructing complex intercellular communication networks (181).

Recent reviews discuss single-cell multi-omics for biomarker development and oncology applications (202, 203). Two-stage multi-view graph spectral clustering offers another computational approach to scRNA-seq analysis (204). These studies provide methodological context; their performance and clinical relevance require separate validation in liver fibrosis.

Future studies will benefit from longitudinal and functional approaches that move beyond descriptive cellular atlases (205). Tracking cellular dynamics during disease progression and regression, particularly in response to therapeutic interventions, will be essential for understanding the reversibility of fibrotic processes (23). Coupling single-cell analyses with experimental perturbation models and lineage-tracing systems may provide direct evidence for the functional roles of specific cell populations and signaling pathways in fibrogenesis (206).

A critical priority is to distinguish single-cell discoveries from clinically validated biomarkers or therapeutic targets. Scar-associated macrophage states marked by genes such as TREM2 and CD9 were resolved at single-cell level and represent disease-associated cell-state candidates, but neither gene is currently established as a stand-alone clinical fibrosis biomarker (23, 63). Similarly, ACTA2 and COL1A1 are canonical fibrogenic/HSC markers that predate scRNA-seq; the added value of single-cell analysis is to localize their expression to specific cell states rather than establish them as novel biomarkers (35). LSEC-associated signatures such as CLEC4G, LYVE1, and RSPO3, together with capillarization-related programs, are mechanistically informative but have not been prospectively validated as diagnostic or prognostic biomarkers (23, 71). Canonical signaling axes such as CCL2–CCR2, TGF-β–TGFβR, and PDGF–PDGFR are supported by prior functional studies; scRNA-seq mainly helps define their potential cellular sources and receivers rather than independently validating them as therapeutic targets (65, 66, 132). Compared with conventional fibrosis assessment, single-cell signatures may provide additional information on cell origin, pathogenic state, and multicellular niche composition; however, their incremental clinical value remains to be established in independent longitudinal cohorts (113, 207, 208).

Complementary preclinical approaches reviewed by Tiwari et al., including CCl_4_- and BDL-induced fibrosis models, HSC cultures, and precision-cut liver slices, can support functional validation of single-cell-derived hypotheses, provided that model selection accounts for the injury mechanism, experimental endpoint, and model-specific limitations (209). Future work should combine such functional validation with spatial and multi-omics profiling in longitudinal human cohorts to determine whether candidate cell states and signaling pathways track disease progression or treatment response, and whether the resulting biomarkers provide additional diagnostic or prognostic value beyond established non-invasive fibrosis tests (207, 208).

## 8 Conclusion

This narrative review integrates single-cell transcriptomic studies of liver fibrogenesis from both animal models and human cohorts, encompassing cellular heterogeneity, trajectory dynamics, intercellular communication networks, and conserved signaling pathways. Through comprehensive analysis of key gene signatures, ligand–receptor interactions, and pathway enrichment, we identify a coordinated remodeling of cellular composition characterized by HSC activation, macrophage accumulation, and LSEC depletion. Furthermore, conserved activation of ECM remodeling, inflammatory signaling, and PI3K–Akt pathways across multiple cell populations is amplified through a central HSC–macrophage communication network. These findings advance our understanding of liver fibrosis mechanisms and inform multicellular-targeted therapies. Future integration of single-cell, spatial omics, and clinical data will further accelerate precision medicine for chronic liver diseases.
