# Supplementary material for: Mechanistic insights and translational perspectives on liver fibrosis through single-cell transcriptomics
Source: Front Immunol. 2026 Oct 27;17:1954491. doi: 10.3389/fimmu.2026.1954491 (PMC13749094; doi:10.3389/fimmu.2026.1954491)
Supplement: Supplementary file 1 [file SupplementaryFile1.docx]

Supplementary Material

# 1 Supplementary Table 1: Changes in cellular proportion before and after liver fibrosis

| Study | Hepatic Stellate Cells (HSCs) | Macrophages | Liver Sinusoidal Endothelial Cells (LSECs) | B lymphocyte |
| --- | --- | --- | --- | --- |
| Yang, 2021  (Mouse, CCl₄/BDL) | Dramatic model-specific reconstruction of activated subcluster composition; highly terminally activated in CCl₄ model, only mildly activated in BDL model. | N.S | N.S | N.S |
|  | CCl₄: |  |  |  |
|  | aHSC became major |  |  |  |
|  | 38.1% stage-2 aHSC (migratory/contractile) |  |  |  |
|  | 43.2% stage-3 aHSC (ECM-producing) |  |  |  |
|  | qHSC sharply decreased |  |  |  |
|  | BDL: |  |  |  |
|  | 44.5% stage-1 aHSC (inflammatory) |  |  |  |
|  | 11.3% stage-2 aHSC |  |  |  |
|  | 11.0% stage-3 aHSC |  |  |  |
|  | qHSC remaining at a certain proportion |  |  |  |
| Cheng, 2023  (Mouse, CCl₄) | Proportion/number significantly increased. | Proportion/number significantly increased. | N.S | N.S |
|  | Quiescent HSC1 nearly disappeared. | Resident Kupffer cells (Mac2) decreased. |  |  |
|  | Activated subclusters (HSC0/HSC2/HSC3) became dominant. | Monocyte-derived macrophages (Mac0/Mac1) became major. |  |  |
| Bai, 2023  (Mouse, TCS) | Proportion/number significantly increased. | LCM_M2 and KC_M2 subtypes increased. | The proportion/number slightly decreased. | Number significantly increased. |
|  | Activated subtypes (aHSC1-6, aHSC_prof) significantly elevated. | Proliferative subtypes enriched. | LSEC_mid2 proportion significantly increased. | B_plasma: 11.95% (≈23-fold increase) |
|  | qHSC: 7.2% (46/638) | M1→M2 polarization | Phenotypic shift to LVEC_central (capillarization). |  |
| Yang, 2023 (Mouse, CCl₄/BDL) | Proportion/number significantly increased. | SAM proportion in hepatic macrophages rises from ~1.0% (normal) to ~13.7% (BDL) and ~13.8% (CCl₄). | N.S | N.S |
| Su, 2021  (Mouse, CCl₄/BDL) | N.S | N.S | The proportion slightly decreased (89% in normal mice → 73% in cirrhotic mice). | N.S |
|  |  |  | Zone 3 (pericentral) LSECs are the most significantly damaged subpopulation. |  |
| Zhang, 2021  (Mouse, CCl₄) | Predominant in quantity, activated from a quiescent state into a heterogeneous population. | N.S | N.S | N.S |
| Ai, 2022  (Human) | Proportion/number significantly increased. | N.S | N.S | N.S |
|  | HSCs differentiate into myofibroblasts. |  |  |  |
| Zhuo, 2025  (Human) | N.S | Proportion/number significantly increased. | N.S | The proportion does not show a significant increase and remains stable overall. |
| Bai, 2023  (Human) | N.S | Macrophage-CD9/IL18 and Macrophage-C1QA proportions increased (p<0.05). | N.S | B cell-IGLC1 proportion decreased (p<0.05). |
|  |  | No significant increase or decrease in other subtypes. |  | No significant difference in the overall proportion of total B cells among immune cells. |
| Ramachandran, 2019 (Human) | PDGFRα+ scar-associated mesenchymal (SAMes) cells, derived from HSCs, were significantly expanded in cirrhotic livers. | Scar-associated macrophages (SAMacs, TREM2+CD9+) expanded markedly. | CD34−CLEC4M+ LSECs reduced in cirrhotic livers. | N.S |
|  | RGS5+ HSCs (quiescent) reduced. | MARCO+TIMD4− Kupffer cells (KC(2)) reduced | Scar-associated endothelia (SAEndo: ACKR1+PLVAP+CD34+) expanded |  |
|  |  | TIMD4+ KCs (KC(1)) unchanged in number. |  |  |

# 2 Supplementary Table 2: Cell differentiation trajectories revealed by single-cell transcriptomic analyses in liver fibrosis

| Study | Disease Model / Tissue | Starting Cell Population | Differentiation Trajectory | Terminal Cell State | Key Marker Genes | Key Signaling Pathways | Trajectory Method |
| --- | --- | --- | --- | --- | --- | --- | --- |
| Yang, 2021 | Mouse (CCl₄/BDL) | Quiescent HSC (qHSC) | qHSC → Stage-1 aHSC (inflammatory) → Stage-2 aHSC (migrative/contractile) → Stage-3 aHSC (ECM-producing) | Stage-3 Activated HSC (myofibroblast) | qHSC: Lrat, Ecm1, Trem56, Angptl6, Vipr1 | Pro-fibrotic: | Slingshot |
|  |  |  |  |  | Stage-1 aHSC: Ccl2, Cxcl10, Cxcl1, Ccl7, Il11 | TGF-β/Smad pathway |  |
|  |  |  |  |  | Stage-2 aHSC: Acta2, Tpm1, Vim, TagIn, Tnc, Tpm2 | PDGF–PDGFRβ pathway |  |
|  |  |  |  |  | Stage-3 aHSC: Col1a1, Col1a2, Col3a1, Lox, Lum | Hippo pathway (Ankrd1, Cyr61, Ctgf, Thbs1) |  |
|  |  |  |  |  |  | NF-κB/STAT3 pathway |  |
|  |  |  |  |  |  | SRF–Fos–Jun pathway |  |
|  |  |  |  |  |  | Anti-fibrotic: |  |
|  |  |  |  |  |  | sGC–cGMP signaling pathway (Gucy1a1/Gucy1b1–NO) |  |
| Yang, 2021 | Mouse (CCl₄/BDL) | Liver-resident Kupffer cell | Kupffer cell → activated Kupffer cell (pro-inflammatory) | Activated Kupffer cell | N.S | Chemokine signaling pathway (CCL2–CCR2) | Slingshot |
|  |  |  |  |  |  | TGF-β/Smad signaling pathway |  |
| Cheng, 2023 | Mouse (CCl₄) | Quiescent HSC (qHSC) | qHSC → HSC0 (myeloid-like myofibroblast) / HSC2 (Clec3b⁺ intermediate activated HSC) / HSC3 (classical myofibroblast) | HSC0 (immunomodulatory aHSC); HSC2 (Clec3b⁺ activated HSC); HSC3 (ECM-producing myofibroblast) | HSC0: Saa3, Slpi, Fth1, Sdc4, Nkain4, Dmkn | Pro-fibrotic: | Monocle |
|  |  |  |  |  | HSC1: Rgs5, Colec11, Reln, Tmem56, Mustn1, Ifitm1 | PI3K–AKT–mTOR pathway (Thbs1-mediated) |  |
|  |  |  |  |  | HSC2: Ebf1, Pi16, Dpt, Ccl11, Clec3b, Penk | TGF-β/Smad pathway |  |
|  |  |  |  |  | HSC3: Tnc, Tagln, Ckb, Acta2, Cxcl14, Col4a1 | PDGF–PDGFRβ pathway |  |
|  |  |  |  |  |  | Functional pathways: |  |
|  |  |  |  |  |  | Immune response pathway (HSC0) |  |
|  |  |  |  |  |  | ECM remodeling pathway (HSC2/HSC3) |  |
|  |  |  |  |  |  | Cell migration and contraction pathway (HSC3) |  |
| Cheng, 2023 | Mouse (CCl₄) | Bone marrow–derived monocyte (Ly6c⁺) | Ly6c⁺ monocyte → Mac1 (profibrotic MDM) → Mac0 (antifibrotic MDM) | Mac0 (Ly6c⁻ antifibrotic macrophage); Mac1 (Ly6c⁺ profibrotic macrophage) | Mac0: Mmp 12, Lpl, Tubb5, Stmn1, 2810417H13Rik, Hist1h2ap | Pro-fibrotic: | Monocle |
|  |  |  |  |  | Mac1: S100a6, S100a4, Thbs1, Chil3, Spp1, Ccl3 | Thbs1–αVβ3/α2Bβ3–PI3K–AKT–mTOR signaling pathway |  |
|  |  |  |  |  |  | TNF-α signaling pathway |  |
|  |  |  |  |  |  | IL-1 signaling pathway |  |
|  |  |  |  |  |  | Anti-fibrotic: |  |
|  |  |  |  |  |  | ECM degradation pathways (Mmp12/Mmp13 mediated) |  |
| Cheng, 2023 | Mouse (CCl₄) | Normal LSEC | Normal LSEC → Mac3 (macrophage–LSEC hybrid cell) | Mac3 (macrophage–LSEC hybrid phenotype) | N.S | N.S | Monocle |
| Bai, 2023 | Mouse (TCS) | Quiescent HSC (qHSC) | qHSC → aHSC1 (ECM-producing) / aHSC4 (chemotactic) / aHSC_prof (proliferative) | aHSC1, aHSC2, aHSC3, aHSC4, aHSC5, aHSC6, aHSC_prof | qHSC: Lrat, Angptl6, Vipr1, Rgs5 | Pro-fibrotic: | monocle(v2.20.0) |
|  |  |  |  |  | aHSC1: Fmod | GAS6–AXL–PI3K–AKT signaling pathway |  |
|  |  |  |  |  | aHSC2: Acta2, Igf1, Col1a1 | TGF-β/Smad signaling pathway |  |
|  |  |  |  |  | aHSC3: Ccl6 | ECM remodeling pathway |  |
|  |  |  |  |  | aHSC4: Ccr2 | Chemotaxis: |  |
|  |  |  |  |  | aHSC5: Ccl6 | Chemokine signaling pathway (mediated by Ccl6, Cxcl10) |  |
|  |  |  |  |  | aHSC6: Thbs1 |  |  |
|  |  |  |  |  | aHSC_prof: Mki67, Mcm6 |  |  |
| Bai, 2023 | Mouse (TCS) | M1 macrophage (LCM_M1 / KC_M1) | M1 macrophage → M2 macrophage (LCM_M2 / KC_M2) / proliferative macrophage (LCM_prof / KC_prof) | LCM_M2, KC_M2, LCM_prof, KC_prof | M1: Cd86, Cd68 | Pro-fibrotic: | monocle(v2.20.0) |
|  |  |  |  |  | M2: Tgfb2, Wnt5a, Mrc1 | IL6–JAK/STAT3 signaling pathway |  |
|  |  |  |  |  | proliferation: Mki67, Stmn1, Cenpf, Ccnb2 | TGF-β/Smad signaling pathway |  |
|  |  |  |  |  |  | T-helper 2 cytokine secretion pathway |  |
|  |  |  |  |  |  | Tissue repair: |  |
|  |  |  |  |  |  | ECM remodeling pathway (Mmp2-mediated) |  |
| Bai, 2023 | Mouse (TCS) | LSEC_pc / LSEC_mid2 | LSEC_pc / LSEC_mid2 → LVEC_central (capillarized endothelial cell) | LVEC_central (capillarized) | LSEC_pc: Kit | Capillarization-related: | monocle(v2.20.0) |
|  |  |  |  |  | LSEC_mid2: Hamp | Wnt signaling pathway |  |
|  |  |  |  |  | LVEC_central: Vwf, Wnt9b, Rspo3 | TGF-β signaling pathway |  |
| Bai, 2023 | Mouse (TCS) | Naïve B cell (B_naive) | B_naive → B_plasma (fibrotic) / B_memory | B_plasma, B_memory | B_naive: Ighd, Fcmr | Fibrosis-related: | monocle(v2.20.0) |
|  |  |  |  |  | B_plasma: Igha, Jchain | ECM organization pathway |  |
|  |  |  |  |  | B_memory: Cd38 | Hypoxia response pathway |  |
|  |  |  |  |  |  | Fibroblast proliferation regulatory pathway |  |
| Yang, 2023 | Mouse (CCl₄/BDL) | Immature Bone Marrow-Derived Macrophage (BMM, Ly6c1⁺Ly6c2⁺) | Immature BMM → Scar-Associated Macrophage (SAM, CD9⁺TREM2⁺) | Scar-Associated Macrophage (SAM) | Immature BMM: Ly6c1, Ly6c2, Ccr2, Itgam | SAM Transformation: | Monocle2 |
|  |  |  |  |  | SAMs(BMM-2/8): Cd9, Trem2, Cd63, Gpnmb, Lgals1, Lgals3 | Plg-Rₖₜ-PLG signaling pathway |  |
|  |  |  |  |  |  | Pro-fibrotic: |  |
|  |  |  |  |  |  | ECM organization pathway |  |
|  |  |  |  |  |  | Collagen biosynthetic pathway (Fn1, Loxl2, Timp2 mediated) |  |
| Su, 2021 | Mouse (CCl₄/BDL) | N.S | N.S | N.S | N.S | N.S | N.S |
| Zhang, 2021 | Mouse (CCl₄) | Activated HSC1 (Early Proliferative) | HSC1 → HSC2 (Mature Myofibroblast) | HSC2, HSC3, HSC4, HSC5, HSC6, HSC7, HSC8 | HSC: Fgl2, Rgs5, Cdc20, Olfml3, PAM, Gm42418, Lmod1, Clec3B | HSC2: | monocle 3 (5) |
|  |  |  | HSC1 → HSC3 (Inflammatory) |  | HSC6: mt-Co3, mt-Atp6, mt-Cytb, mt-Co1, mt-Co2, mt-Nd4, mt-Nd1, Hspa1b, Hspa1a | Cellular contraction pathways |  |
|  |  |  | HSC1 → HSC4 (Vasoactive/Repair) → HSC7 (VSMC-like) |  |  | Collagen assembly pathways |  |
|  |  |  | HSC1 → HSC8 (Fibroblast-like) |  |  | Platelet activation pathways |  |
|  |  |  | HSC2 → HSC5 (Axon Growth-Associated) |  |  | HSC3: |  |
|  |  |  | HSC4 → HSC6 (Metabolic Stress/Apoptotic) |  |  | Chemokine signaling pathways |  |
|  |  |  |  |  |  | ECM organization pathway |  |
|  |  |  |  |  |  | Collagen biosynthesis pathways |  |
|  |  |  |  |  |  | HSC4: |  |
|  |  |  |  |  |  | Complement signaling pathways |  |
|  |  |  |  |  |  | Immune interaction pathways |  |
|  |  |  |  |  |  | Angiogenesis pathways |  |
|  |  |  |  |  |  | HSC5: |  |
|  |  |  |  |  |  | FGFR signaling pathways |  |
|  |  |  |  |  |  | L1CAM interaction pathways |  |
|  |  |  |  |  |  | Axon guidance pathways |  |
|  |  |  |  |  |  | HSC8: |  |
|  |  |  |  |  |  | Tissue repair pathways |  |
|  |  |  |  |  |  | Wound healing pathways |  |
| Ai, 2022 | Human | Quiescent HSC (C1 cluster) | Quiescent HSC → Myofibroblast (C2 cluster) | Myofibroblast | HSCs: RGS5, ACTA2, PDGFRB, ADIRF | Pro-fibrotic: | monocle3 (v1.0) |
|  |  |  |  |  | Myofibroblasts(MFBs): RBP1, COL1A1, COL6A3, COL3A1 | PDGF signaling pathway (PDGFA-PDGFRA) |  |
|  |  |  |  |  |  | GAS6-AXL signaling pathway |  |
|  |  |  |  |  |  | MDK-LRP1 signaling pathway |  |
|  |  |  |  |  |  | Notch signaling pathway (JAG1-NOTCH) |  |
|  |  |  |  |  |  | ECM deposition pathway (COL1A1/COL3A1/COL6A3 mediated) |  |
| Zhuo, 2025 | Human | Non-classical monocyte (C6 cluster, CD14⁺CD16⁺⁺) | Non-classical monocyte → intermediate monocyte (C5 cluster, CD14⁺⁺CD16⁺) → classical monocyte (C1–C3 clusters, CD14⁺⁺CD16⁻), including monocyte–platelet aggregate (MPA, C4 cluster, CD14⁺⁺CD16⁻CD41⁺) | Classical monocyte (C1–C3), monocyte–platelet aggregate (MPA, C4), intermediate monocyte (C5) | Monocyte: CD14^+^, CD16^+^ | Pro-inflammatory: | Monocle 3 |
|  |  |  |  |  |  | Chemokine signaling pathway (CCL5–CCR1/CCR5) |  |
|  |  |  |  |  |  | TNF signaling pathway |  |
|  |  |  |  |  |  | IL-17 signaling pathway |  |
|  |  |  |  |  |  | Fibrosis-related: |  |
|  |  |  |  |  |  | PDGF signaling pathway (PDGFA–PDGFRβ) |  |
|  |  |  |  |  |  | Adhesion: |  |
|  |  |  |  |  |  | Focal Adhesion Pathway |  |
|  |  |  |  |  |  | Leukocyte Transendothelial Migration Pathway |  |
| Zhuo, 2025 | Human | Naïve B cell (CD79A⁺) | Naïve B cell → plasma B cell (Jchain⁺) | Plasma B cell | Naïve B cell: CD79A | B cell receptor signaling pathway (implied from B cell lineage markers) | Monocle 3 |
|  |  |  |  |  | Plasma B cell: JCHAIN |  |  |
| Bai, 2023 | Human | Immature Monocytes (Monocyte-S100A6, Monocyte-S100A9, Monocyte-FCGR3A) | Immature Monocytes → Macrophage subsets (Macrophage-CD9/IL18, Macrophage-C1QA, Macrophage-CCL5, etc.) / Dendritic Cells (cDC1, cDC2, pDC) | Macrophage-CD9/IL18, Macrophage-C1QA, Macrophage-CCL5, Macrophage-PLCG2, Macrophage-NAMPT, cDC1, cDC2, pDC | Immature Monocytes: S100A6, S100A9, FCGR3A (CD16), CD14 | Angiogenesis | monocle 2.22.0 |
|  |  |  |  |  | Macrophage-CD9/IL18: CD9, IL18 | Extracelluar matrix organization |  |
|  |  |  |  |  | Macrophage-C1QA: C1QA, C1QB, C1QC |  |  |
|  |  |  |  |  | Terminal Macrophages: PLCG2, NAMPT |  |  |
|  |  |  |  |  | Dendritic Cells: HLA-DQA1, HLA-DPA1 |  |  |
| Bai, 2023 | Human | Naive B Cell | Naive B Cell → Mature B Cell subsets (B cell-IGLC1, B cell-FCRL5, etc.) | B cell-IGLC1, B cell-FCRL5, B cell-PLCG2, B cell-TNFSF9 | Naive B Cell: Ighd, CD79A, MS4A1 | B Cell receptor signaling pathway | monocle 2.22.0 |
|  |  |  |  |  | B cell-IGLC1: IGLC1, IGLC2 | Immune response pathway (GO/KEGG enrichment) |  |
|  |  |  |  |  | Mature B Cells: FCRL5, TNFSF9, PLCG2 |  |  |
| Ramachandran, 2019 | Human | Circulating monocytes (blood monocytes) | Circulating monocytes → tissue monocytes (TMs) → scar-associated macrophages (SAMacs, TREM2⁺CD9⁺) / conventional dendritic cells (cDCs) | SAMac(1), SAMac(2), cDCs | cDCsCirculating Monocytes: MNDA, S100A12 | Pro-fibrogenic pathways (SPP1, LGALS3, CCL2, PDGFB mediated) | Monocle(v.2.6.1) |
|  |  |  |  |  | Tissue Monocytes: CD14, CD16, CCR2 | TNFRSF12A signaling pathway |  |
|  |  |  |  |  | SAMacs: TREM2, CD9, IL1B, SPP1, LGALS3, TNFSF12 | Chemokine signaling pathway |  |
|  |  |  |  |  | cDCs: CD1C, CLEC9A |  |  |
| Ramachandran, 2019 | Human | Quiescent HSC (qHSC) | Quiescent HSC → scar-associated mesenchymal cells (SAMes, PDGFRA⁺) | Scar-associated mesenchymal cells (SAMes) | Quiescent HSC: RGS5, IGFBP5, ADAMTS1, GEM | ECM deposition pathway | Monocle(v.2.6.1) |
|  |  |  |  |  | SAMes: PDGFRα, COL1A1, COL1A2, COL3A1, TIMP1 | PDGF signaling pathway |  |
|  |  |  |  |  |  | NOTCH signaling pathway |  |
| Ramachandran, 2019 | Human | Normal LSEC (CD34⁻CLEC4M⁺) | Normal LSEC → scar-associated endothelia [SAEndo(1): CD34⁺PLVAP⁺VWA1⁺; SAEndo(2): CD34⁺PLVAP⁺ACKR1⁺] | Scar-associated endothelia (SAEndo1, SAEndo2) | Normal LSEC: CLEC4M, CLEC4G, CD34^-^ | Notch signaling pathway (JAG1/JAG2/DLL4–NOTCH3) | Monocle(v.2.6.1) |
|  |  |  |  |  | SAEndo(1): PLVAP, CD34, VWA1, PDGFB, PDGFD | PDGF signaling pathway (PDGFB/PDGFD-mediated) |  |
|  |  |  |  |  | SAEndo(2): PLVAP, CD34, ACKR1 | Leukocyte transmigration pathway (ACKR1-mediated) |  |

# 3 Supplementary Table 3: Cell–cell communication networks identified by single-cell transcriptomic analyses in liver fibrosis

| Study | Disease Model / Tissue | Sender | Receiver | Ligand–Receptor | Pathway |
| --- | --- | --- | --- | --- | --- |
| Yang, 2021 | Mouse (CCl₄/BDL) | Hepatic Stellate Cell (aHSC, Stage1) | Macrophage (Kupffer Cell) | CCL2-CCR2 | Chemokine Signaling Pathway |
| Yang, 2021 | Mouse (CCl₄/BDL) | Hepatic Stellate Cell (aHSC, Stage1) | Macrophage (Kupffer Cell) | CXCL10-CXCR3 | Chemokine Signaling Pathway |
| Yang, 2021 | Mouse (CCl₄/BDL) | Hepatic Stellate Cell (aHSC, Stage1) | Macrophage (Kupffer Cell) | CXCL1-CXCR1 | Chemokine Signaling Pathway |
| Yang, 2021 | Mouse (CCl₄/BDL) | Hepatic Stellate Cell (aHSC, Stage1) | Macrophage (Kupffer Cell) | CCL7-CCR1 | Chemokine Signaling Pathway |
| Yang, 2021 | Mouse (CCl₄/BDL) | Hepatic Stellate Cell (aHSC, Stage1) | Macrophage (Kupffer Cell) | CCL2-CCR2 | NF-κB/STAT3 Transcription Factor Pathway |
| Yang, 2021 | Mouse (CCl₄/BDL) | Hepatic Stellate Cell (aHSC, Stage1) | Macrophage (Kupffer Cell) | CXCL10-CXCR3 | NF-κB/STAT3 Transcription Factor Pathway |
| Yang, 2021 | Mouse (CCl₄/BDL) | Hepatic Stellate Cell (aHSC, Stage1) | Macrophage (Kupffer Cell) | CXCL1-CXCR1 | NF-κB/STAT3 Transcription Factor Pathway |
| Yang, 2021 | Mouse (CCl₄/BDL) | Hepatic Stellate Cell (aHSC, Stage1) | Macrophage (Kupffer Cell) | CCL7-CCR1 | NF-κB/STAT3 Transcription Factor Pathway |
| Yang, 2021 | Mouse (CCl₄/BDL) | Hepatic Stellate Cell (aHSC, Stage1) | Hepatic Stellate Cell (aHSC) | CCL2-CCR2 | Chemokine Signaling Pathway |
| Yang, 2021 | Mouse (CCl₄/BDL) | Liver Sinusoidal Endothelial Cell (LSEC) | Hepatic Stellate Cell (qHSC primarily) | NO (Nitric Oxide)-Gucy1a1/Gucy1b1 (sGC α1/β1 Subunits) | sGC-cGMP Pathway |
| Yang, 2021 | Mouse (CCl₄/BDL) | Macrophage (Kupffer Cell) | Hepatic Stellate Cell (qHSC→aHSC) | TGFβ-TGFβRⅠ/Ⅱ | TGFβ-Smad Pathway |
| Yang, 2021 | Mouse (CCl₄/BDL) | Macrophage (Kupffer Cell) | Hepatic Stellate Cell (qHSC→aHSC) | CCL2-CCR2 | Chemokine Signaling Pathway |
| Yang, 2021 | Mouse (CCl₄/BDL) | Hepatic Stellate Cell (aHSC) | Hepatic Stellate Cell (aHSC) | PDGF-PDGFRβ | PDGF-PDGFRβ Pathway |
| Yang, 2021 | Mouse (CCl₄/BDL) | Hepatic Stellate Cell (aHSC) | Hepatic Stellate Cell (aHSC) | TGFβ-TGFβRⅠ/Ⅱ | TGFβ-Smad Pathway |
| Yang, 2021 | Mouse (CCl₄/BDL) | Hepatic Stellate Cell (aHSC) | Hepatic Stellate Cell (aHSC) | Ankrd1 (Hippo pathway target) | Hippo Signaling Pathway |
| Yang, 2021 | Mouse (CCl₄/BDL) | Hepatic Stellate Cell (aHSC) | Hepatic Stellate Cell (aHSC) | Cyr61 (Hippo pathway target) | Hippo Signaling Pathway |
| Yang, 2021 | Mouse (CCl₄/BDL) | Hepatic Stellate Cell (aHSC) | Hepatic Stellate Cell (aHSC) | Ctgf (Hippo pathway target) | Hippo Signaling Pathway |
| Yang, 2021 | Mouse (CCl₄/BDL) | Hepatic Stellate Cell (aHSC) | Hepatic Stellate Cell (aHSC) | Thbs1 (Hippo pathway target) | Hippo Signaling Pathway |
| Cheng, 2023 | Mouse (CCl₄) | Macrophage (Mac1, Ly6c⁺ MDMs) | Hepatic Stellate Cell (HSC0, HSC3) | Thbs1–αVβ3 (Itgb3) | PI3K/AKT/mTOR Signaling Pathway |
| Cheng, 2023 | Mouse (CCl₄) | Macrophage (Mac1, Ly6c⁺ MDMs) | Hepatic Stellate Cell (HSC0, HSC3) | Thbs1–α2Bβ3 (Itga2b&Itgb3) | PI3K/AKT/mTOR Signaling Pathway |
| Cheng, 2023 | Mouse (CCl₄) | Macrophage | Hepatic Stellate Cell | PDGF–PDGFRβ | PDGF-PDGFRβ Signaling Pathway |
| Cheng, 2023 | Mouse (CCl₄) | Macrophage | Hepatic Stellate Cell | TGFβ1–TGFβRⅠ/Ⅱ | TGFβ-Smad Signaling Pathway |
| Cheng, 2023 | Mouse (CCl₄) | Macrophage | Hepatic Stellate Cell | IGF-1–IGF-1R (Implied) | IGF-1 Signaling Pathway |
| Cheng, 2023 | Mouse (CCl₄) | Macrophage | Hepatic Stellate Cell | VEGF–VEGFR (Implied) | VEGF Signaling Pathway |
| Cheng, 2023 | Mouse (CCl₄) | Macrophage | Hepatic Stellate Cell | Timp1–MMPs (Implied) | ECM Remodeling Pathway |
| Cheng, 2023 | Mouse (CCl₄) | Macrophage | Hepatic Stellate Cell | Osm–OsmR (Implied) | Osm Signaling Pathway |
| Cheng, 2023 | Mouse (CCl₄) | Macrophage | Hepatic Stellate Cell | TNF–TNFR | TNF-α Signaling Pathway |
| Cheng, 2023 | Mouse (CCl₄) | Macrophage | Hepatic Stellate Cell | IL1 family–IL1R | IL1 Signaling Pathway |
| Cheng, 2023 | Mouse (CCl₄) | Macrophage | Hepatic Stellate Cell | Lgals9–Lgals9 Receptor (Implied) | Lgals9 Signaling Pathway |
| Cheng, 2023 | Mouse (CCl₄) | Macrophage | Hepatic Stellate Cell | CCL3–CCR1/5 (Implied) | Chemokine Signaling Pathway |
| Cheng, 2023 | Mouse (CCl₄) | Macrophage | Hepatic Stellate Cell | TNFSF12–TNFRSF12A | TNFSF12-TNFRSF12A Signaling Pathway |
| Cheng, 2023 | Mouse (CCl₄) | Macrophage | Hepatic Stellate Cell | Sema4d–PlexinD1/Neuropilin (Implied) | Sema4d Signaling Pathway |
| Cheng, 2023 | Mouse (CCl₄) | Macrophage | Hepatic Stellate Cell | Fn1–Integrin Receptors (Implied) | Fn1-Integrin Signaling Pathway |
| Cheng, 2023 | Mouse (CCl₄) | Hepatic Stellate Cell (Activated) | Macrophage | Spp1–CD44/Osteopontin Receptor (Implied) | Spp1 Signaling Pathway |
| Cheng, 2023 | Mouse (CCl₄) | Hepatic Stellate Cell (Activated) | Macrophage | TNFSF12–TNFRSF12A | TNFSF12-TNFRSF12A Signaling Pathway |
| Cheng, 2023 | Mouse (CCl₄) | Hepatic Stellate Cell (Activated) | Macrophage | Fn1–Integrin Receptors (Implied) | Fn1-Integrin Signaling Pathway |
| Cheng, 2023 | Mouse (CCl₄) | Liver Sinusoidal Endothelial Cell (LSEC) | Hepatic Stellate Cell | Notch1–JAG1 (Implied) | Notch Signaling Pathway |
| Bai, 2023 | Mouse (TCS) | Liver Sinusoidal Endothelial Cell (LSEC_mid2) | Hepatic Stellate Cell (aHSC1) | GAS6–AXL | PI3K/AKT Signaling Pathway |
| Bai, 2023 | Mouse (TCS) | Macrophage (KC_M2/LCM_M2) | Hepatic Stellate Cell (aHSC1) | GAS6–AXL | PI3K/AKT Signaling Pathway |
| Bai, 2023 | Mouse (TCS) | Macrophage (KC_M2/LCM_M2) | Hepatic Stellate Cell (aHSC1) | GAS6–MERTK | Pro-fibrotic Signaling Pathway |
| Bai, 2023 | Mouse (TCS) | Macrophage (KC_M2) | Hepatic Stellate Cell (aHSC1) | IL6–IL6 Receptor | IL6/JAK-STAT3 Signaling Pathway |
| Bai, 2023 | Mouse (TCS) | Macrophage (KC_M2/LCM_M2) | Hepatic Stellate Cell (aHSC1) | TGFB2–TGFβ Receptor2/3 | TGFβ-Smad Signaling Pathway |
| Bai, 2023 | Mouse (TCS) | Macrophage (KC_M2/LCM_M2) | Hepatic Stellate Cell (aHSC1) | TGFB3–TGFβ Receptor2/3 | TGFβ-Smad Signaling Pathway |
| Bai, 2023 | Mouse (TCS) | Macrophage (KC_M2/LCM_M2) | Hepatic Stellate Cell (aHSC1) | TNF–TNFRSF1A | TNF-α Signaling Pathway |
| Bai, 2023 | Mouse (TCS) | Liver Sinusoidal Endothelial Cell (LSEC_mid2) | Hepatic Stellate Cell (aHSC1) | NOTCH1–WNT4 | Notch/WNT Signaling Pathway |
| Bai, 2023 | Mouse (TCS) | Hepatic Stellate Cell (aHSC1) | Macrophage (KC_M2/LCM_M2) | Collagen Molecules–Integrin Complex | ECM-Integrin Signaling Pathway |
| Bai, 2023 | Mouse (TCS) | B Lymphocyte (B_plasma) | Hepatic Stellate Cell (aHSC1) | Collagen Molecules–Integrin Complex | ECM-Integrin Signaling Pathway |
| Bai, 2023 | Mouse (TCS) | Macrophage (LCM_M2) | Hepatic Stellate Cell (aHSC1) | CCL2–CCR2 | Chemokine Signaling Pathway |
| Bai, 2023 | Mouse (TCS) | Macrophage (LCM_M2) | Hepatic Stellate Cell (aHSC1) | IL1B–IL1 Receptor | IL1 Signaling Pathway |
| Bai, 2023 | Mouse (TCS) | Macrophage (KC_M2) | Hepatic Stellate Cell (aHSC1) | MMP2–ECM Receptor | ECM Remodeling Signaling Pathway |
| Yang, 2023 | Mouse (CCl₄/BDL) | Bone Marrow-Derived Macrophage (BMM) | Scar-Associated Macrophage (SAM) | PLG–Plg-Rₖₜ | SAM Phenotypic Transformation Pathway |
| Yang, 2023 | Mouse (CCl₄/BDL) | Scar-Associated Macrophage (SAM) | Hepatic Stellate Cell (aHSC) | Fn1–Integrin Receptors (Implied) | ECM Remodeling Signaling Pathway |
| Yang, 2023 | Mouse (CCl₄/BDL) | Scar-Associated Macrophage (SAM) | Hepatic Stellate Cell (aHSC) | Spp1–CD44/Osteopontin Receptor (Implied) | Pro-Fibrotic Signaling Pathway |
| Yang, 2023 | Mouse (CCl₄/BDL) | Scar-Associated Macrophage (SAM) | Hepatic Stellate Cell (aHSC) | Loxl2–Lysyl Oxidase Receptor (Implied) | Collagen Crosslinking Pathway |
| Yang, 2023 | Mouse (CCl₄/BDL) | Scar-Associated Macrophage (SAM) | Hepatic Stellate Cell (aHSC) | Timp2–MMPs (Implied) | ECM Homeostasis Pathway |
| Yang, 2023 | Mouse (CCl₄/BDL) | Scar-Associated Macrophage (SAM) | Hepatic Stellate Cell (aHSC) | Ctsb/Ctsd/Ctsl–Cathepsin Receptors (Implied) | Collagen Degradation/Remodeling Pathway |
| Yang, 2023 | Mouse (CCl₄/BDL) | Scar-Associated Macrophage (SAM) | Hepatic Stellate Cell (aHSC) | Anxa2–Integrin αVβ3 (Implied) | Cell Adhesion/Activation Pathway |
| Yang, 2023 | Mouse (CCl₄/BDL) | Scar-Associated Macrophage (SAM) | Hepatic Stellate Cell (aHSC) | Vim–Vimentin Receptor (Implied) | Cytoskeleton Remodeling Pathway |
| Yang, 2023 | Mouse (CCl₄/BDL) | Scar-Associated Macrophage (SAM) | Hepatic Stellate Cell (aHSC) | Ccl2–CCR2 | Chemokine Signaling Pathway |
| Yang, 2023 | Mouse (CCl₄/BDL) | Scar-Associated Macrophage (SAM) | Hepatic Stellate Cell (aHSC) | Ccl7–CCR1/5 (Implied) | Chemokine Signaling Pathway |
| Su, 2021 | Mouse (CCl₄/BDL) | Liver Sinusoidal Endothelial Cell (Zone 1 LSEC, EC2) | Hepatic Stellate Cell (qHSC) | NO–Gucy1a1/Gucy1b1 (Implied) | eNOS-NO Signaling Pathway |
| Su, 2021 | Mouse (CCl₄/BDL) | Liver Sinusoidal Endothelial Cell (Zone 2 LSEC, EC3) | Hepatic Stellate Cell (qHSC) | NO–Gucy1a1/Gucy1b1 (Implied) | eNOS-NO Signaling Pathway |
| Su, 2021 | Mouse (CCl₄/BDL) | Liver Sinusoidal Endothelial Cell (Zone 3 LSEC, EC4) | Hepatic Stellate Cell (qHSC) | NO–Gucy1a1/Gucy1b1 (Implied) | eNOS-NO Signaling Pathway |
| Su, 2021 | Mouse (CCl₄/BDL) | Liver Sinusoidal Endothelial Cell (All zones LSEC) | Hepatic Stellate Cell (aHSC) | CD34–Integrin Receptors (Implied) | LSEC Capillarization-Associated Pathway |
| Su, 2021 | Mouse (CCl₄/BDL) | Liver Sinusoidal Endothelial Cell (All zones LSEC) | Hepatic Stellate Cell (aHSC) | Col4a1/Col4a2/Col5a2–Integrin Receptors (Implied) | ECM Deposition Pathway |
| Su, 2021 | Mouse (CCl₄/BDL) | Liver Sinusoidal Endothelial Cell (All zones LSEC) | Macrophage | Ccl2–CCR2 (Implied) | Chemokine Signaling Pathway |
| Su, 2021 | Mouse (CCl₄/BDL) | Macrophage (Kupffer Cell/BMM) | Liver Sinusoidal Endothelial Cell (All zones LSEC) | TNF–TNFR | TNF Signaling Pathway |
| Su, 2021 | Mouse (CCl₄/BDL) | Macrophage (Kupffer Cell/BMM) | Liver Sinusoidal Endothelial Cell (All zones LSEC) | IL17–IL17 Receptor | IL17 Signaling Pathway |
| Su, 2021 | Mouse (CCl₄/BDL) | Scar-Associated Macrophage (SAM) | Hepatic Stellate Cell (aHSC) | Fn1–Integrin Receptors (Implied) | ECM Remodeling Pathway |
| Su, 2021 | Mouse (CCl₄/BDL) | Scar-Associated Macrophage (SAM) | Hepatic Stellate Cell (aHSC) | Loxl2–Lysyl Oxidase Receptor (Implied) | Collagen Crosslinking Pathway |
| Su, 2021 | Mouse (CCl₄/BDL) | Bone Marrow-Derived Macrophage (BMM) | Scar-Associated Macrophage (SAM) | PLG–Plg-Rₖₜ | SAM Phenotypic Transformation Pathway |
| Zhang, 2021 | Mouse (CCl₄) | Hepatic Stellate Cell (HSC1, Early Activated) | Hepatic Stellate Cell (HSC2) | Cdc20–Cyclin B1 (Implied) | Mitotic Cell Cycle Pathway |
| Zhang, 2021 | Mouse (CCl₄) | Hepatic Stellate Cell (HSC1, Early Activated) | Hepatic Stellate Cell (HSC3) | Cdc20–Cyclin B1 (Implied) | Mitotic Cell Cycle Pathway |
| Zhang, 2021 | Mouse (CCl₄) | Hepatic Stellate Cell (HSC1, Early Activated) | Hepatic Stellate Cell (HSC4) | Cdc20–Cyclin B1 (Implied) | Mitotic Cell Cycle Pathway |
| Zhang, 2021 | Mouse (CCl₄) | Hepatic Stellate Cell (HSC2, Mature Myofibroblast) | Macrophage | Fgl2–FcγR (Implied) | Immune Tolerance Pathway |
| Zhang, 2021 | Mouse (CCl₄) | Hepatic Stellate Cell (HSC3, Inflammatory) | Macrophage | CCL2–CCR2 | Chemokine Signaling Pathway |
| Zhang, 2021 | Mouse (CCl₄) | Hepatic Stellate Cell (HSC3, Inflammatory) | Macrophage | CCL7–CCR1/5 | Chemokine Signaling Pathway |
| Zhang, 2021 | Mouse (CCl₄) | Hepatic Stellate Cell (HSC3, Inflammatory) | Neutrophil (Implied) | CXCL1–CXCR1/2 | Chemokine Signaling Pathway |
| Zhang, 2021 | Mouse (CCl₄) | Hepatic Stellate Cell (HSC3, Inflammatory) | Neutrophil (Implied) | CXCL2–CXCR1/2 | Chemokine Signaling Pathway |
| Zhang, 2021 | Mouse (CCl₄) | Hepatic Stellate Cell (HSC4, Vasoactive/Repair) | Liver Sinusoidal Endothelial Cell (LSEC) | Angptl6–Tie2 (Implied) | Angiogenesis Pathway |
| Zhang, 2021 | Mouse (CCl₄) | Hepatic Stellate Cell (HSC4, Vasoactive/Repair) | Liver Sinusoidal Endothelial Cell (LSEC) | Rgs5–Gα₁₂/₁₃ (Implied) | Vasomotor Regulation Pathway |
| Zhang, 2021 | Mouse (CCl₄) | Liver Sinusoidal Endothelial Cell (LSEC) | Hepatic Stellate Cell (qHSC/aHSC) | Clec4g–Unknown Receptor (Implied) | LSEC–HSC Crosstalk Pathway |
| Zhang, 2021 | Mouse (CCl₄) | Macrophage (Kupffer Cell) | Hepatic Stellate Cell (aHSC) | C1qc–Complement Receptor (Implied) | Complement Signaling Pathway |
| Ai, 2022 | Human | Hepatic Stellate Cell (HSC, C1 cluster) | Myofibroblast (C2 cluster) | PDGFA–PDGFRA | PDGF Signaling Pathway |
| Ai, 2022 | Human | Hepatic Stellate Cell (HSC, C1 cluster) | Myofibroblast (C2 cluster) | GAS6–AXL (Implied) | GAS6–AXL Signaling Pathway |
| Ai, 2022 | Human | Hepatic Stellate Cell (HSC, C1 cluster) | Myofibroblast (C2 cluster) | MDK–LRP1 | MDK–LRP1 Signaling Pathway |
| Ai, 2022 | Human | Hepatic Stellate Cell (HSC, C1 cluster) | Myofibroblast (C2 cluster) | AGT–AGTR1 (Implied) | Angiotensin Signaling Pathway |
| Ai, 2022 | Human | Hepatic Stellate Cell (HSC, C1 cluster) | Myofibroblast (C2 cluster) | PGF–FLT1 (Implied) | VEGF/PGF Signaling Pathway |
| Ai, 2022 | Human | Hepatic Stellate Cell (HSC, C1 cluster) | Myofibroblast (C2 cluster) | NTF3–TRKC (Implied) | Neurotrophin Signaling Pathway |
| Ai, 2022 | Human | Hepatic Stellate Cell (HSC, C1 cluster) | Myofibroblast (C2 cluster) | JAG1–NOTCH (Implied) | Notch Signaling Pathway |
| Ai, 2022 | Human | Hepatic Stellate Cell (HSC, C1 cluster) | Myofibroblast (C2 cluster) | PROS1–TYRO3 (Implied) | TAM Receptor Signaling Pathway |
| Zhuo, 2025 | Human | Monocyte–Platelet Aggregate (MPA, CD14⁺⁺CD16⁻CD41⁺) | Hepatic Stellate Cell (HSC, LX-2 cell line) | CCL5–CCR1/5 (Implied) | Chemokine Signaling Pathway |
| Zhuo, 2025 | Human | Monocyte–Platelet Aggregate (MPA, CD14⁺⁺CD16⁻CD41⁺) | Hepatic Stellate Cell (HSC, LX-2 cell line) | PDGF-A–PDGFRβ (Implied) | PDGF Signaling Pathway |
| Zhuo, 2025 | Human | Monocyte–Platelet Aggregate (MPA, CD14⁺⁺CD16⁻CD41⁺) | Liver Sinusoidal Endothelial Cell (LSEC, HuVec) | Integrin–ECM Receptors (Implied) | Focal Adhesion Pathway |
| Bai, 2023 | Human | Macrophage (CD9⁺/IL18⁺ subset) | Hepatic Stellate Cell (aHSC) | IL18–IL18R1 / IL18RAP | IL18 Signaling Pathway |
| Bai, 2023 | Human | Macrophage (C1QA⁺ subset) | Hepatic Stellate Cell (aHSC) | C1QA–Complement Receptor | Complement Signaling Pathway |
| Bai, 2023 | Human | Macrophage (CCL5⁺ subset) | Hepatic Stellate Cell (aHSC) | CCL5–CCR1 / CCR5 | Chemokine Signaling Pathway |
| Bai, 2023 | Human | Macrophage (THBS1⁺ subset) | Hepatic Stellate Cell (aHSC) | THBS1–Integrin Receptors (αVβ3 etc.) | ECM–Integrin Signaling Pathway |
| Bai, 2023 | Human | Macrophage (CD9⁺/IL18⁺ subset) | B Lymphocyte (IGLC1⁺ B cell) | CD9–Unknown Receptor (Implied) | Cell Adhesion–Mediated Signaling |
| Bai, 2023 | Human | Monocyte / Macrophage (S100A9⁺) | B Lymphocyte (IGLC1⁺ B cell) | S100A9–RAGE | S100A9–RAGE Signaling Pathway |
| Bai, 2023 | Human | B Lymphocyte (IGLC1⁺ B cell) | Macrophage (C1QA⁺ subset) | IGLC1–FcγR | FcγR-Mediated Signaling |
| Bai, 2023 | Human | B Lymphocyte (IGLC1⁺ B cell) | Hepatic Stellate Cell (aHSC) | IGLC1–Integrin Complex | ECM-Associated Signaling |
| Bai, 2023 | Human | Liver Sinusoidal Endothelial Cell (LSEC) | Macrophage (CD9⁺/IL18⁺ subset) | CCL2–CCR2 | Chemokine Signaling Pathway |
| Bai, 2023 | Human | Liver Sinusoidal Endothelial Cell (LSEC) | Hepatic Stellate Cell (qHSC) | NO–GUCY1A1 / GUCY1B1 (sGC) | eNOS–NO–sGC Signaling Pathway |
| Bai, 2023 | Human | Hepatic Stellate Cell (aHSC) | Macrophage (CCL5⁺ subset) | CCL2–CCR2 | Chemokine Signaling Pathway |
| Bai, 2023 | Human | Hepatic Stellate Cell (aHSC) | Macrophage (CD9⁺/IL18⁺ subset) | TNF–TNFR1 / TNFR2 | TNF-α Signaling Pathway |
| Ramachandran, 2019 | Human | Macrophage (Scar-Associated Macrophage, SAMac) | Hepatic Stellate Cell (HSC) / Scar-Associated Mesenchymal Cell (SAMes) | TNFSF12–TNFRSF12A | TNFRSF12A Signaling Pathway |
| Ramachandran, 2019 | Human | Macrophage (SAMac) | HSC / SAMes | PDGFB–PDGFRA | PDGF Signaling Pathway |
| Ramachandran, 2019 | Human | Macrophage (SAMac) | HSC / SAMes | EGFR ligands (unspecified)–EGFR | EGFR Signaling Pathway |
| Ramachandran, 2019 | Human | Macrophage (SAMac) | HSC / SAMes | CCL2–CCR2 | Chemokine Signaling Pathway |
| Ramachandran, 2019 | Human | Macrophage (SAMac) | HSC / SAMes | SPP1–Integrin receptors | ECM–Integrin Signaling Pathway |
| Ramachandran, 2019 | Human | Macrophage (SAMac) | HSC / SAMes | LGALS3–Galectin receptors | Galectin Signaling Pathway |
| Ramachandran, 2019 | Human | Liver Sinusoidal Endothelial Cell (LSEC) / Scar-Associated Endothelium (SAEndo) | HSC / SAMes | JAG1–NOTCH3 | Notch Signaling Pathway |
| Ramachandran, 2019 | Human | Liver Sinusoidal Endothelial Cell (LSEC) / SAEndo | HSC / SAMes | JAG2–NOTCH3 | Notch Signaling Pathway |
| Ramachandran, 2019 | Human | Liver Sinusoidal Endothelial Cell (LSEC) / SAEndo | HSC / SAMes | DLL4–NOTCH3 | Notch Signaling Pathway |
| Ramachandran, 2019 | Human | Liver Sinusoidal Endothelial Cell (LSEC) / SAEndo | HSC / SAMes | PDGFB–PDGFRA | PDGF Signaling Pathway |
| Ramachandran, 2019 | Human | Liver Sinusoidal Endothelial Cell (LSEC) / SAEndo | HSC / SAMes | PDGFD–PDGFRβ | PDGF Signaling Pathway |
| Ramachandran, 2019 | Human | HSC / SAMes | Macrophage (SAMac) | CSF1–CSF1R | CSF1–CSF1R Signaling Pathway |

Supplementary Figure 1. Literature search and study selection. Records were identified through Google Scholar, PubMed, and Web of Science for the publication period from 1 January 2015 to 31 December 2025. After merging the records, EndNote deduplication, research-article filtering, title and abstract screening, and eligibility assessment yielded 10 articles: six animal studies and four human studies. Exclusions are shown as branches from the main flow. The flow structure was adapted from PRISMA 2020 to reflect the screening sequence. Counts refer to records or articles rather than independent cohorts.
